# Migration and non-breeding ecology of the Yellow-breasted Chat *Icteria virens*

**DOI:** 10.1007/s10336-021-01931-8

**Published:** 2021-10-15

**Authors:** Kristen A. Mancuso, Karen E. Hodges, John D. Alexander, Manuel Grosselet, A. Michael Bezener, Luis Morales, Sarahy C. Martinez, Jessica Castellanos-Labarcena, Michael A. Russello, Sarah M. Rockwell, Matthias E. Bieber, Christine A. Bishop

**Affiliations:** 1 Department of Biology, University of British Columbia, Okanagan Campus, Kelowna, BC Canada; 2 Klamath Bird Observatory, Ashland, OR USA; 3 Tierra de Aves, Veracruz, Mexico; 4 En’owkin Center, Penticton, BC Canada; 5 Observatorio de Aves de San Pancho, San Francisco, Nayarit Mexico; 6 Universidad de Guadalajara-CUCSUR, Jalisco, Mexico; 7 Department of Integrative Biology, University of Guelph, Guelph, ON Canada; 8 Science and Technology Branch, Environment and Climate Change Canada, Delta, BC Canada

**Keywords:** *Icteria virens*, Migratory connectivity, GPS tracking, Annual cycle, Neotropical migrant

## Abstract

Detailed information spanning the full annual cycle is lacking for most songbird populations. We examined breeding, migration, and non-breeding sites for the Yellow-breasted Chat (*Icteria virens,* chat). We deployed archival GPS tags and light-level geolocators on breeding chats in British Columbia and light-level geolocators in California from 2013 to 2017 to determine migration routes and non-breeding sites. We examined whether chats overwintered in protected areas and characterized the percent of land cover within 1 km. We used a combination of genetics and stable hydrogen isotopes from feathers collected on non-breeding chats in Nayarit, Mexico (2017–2019) and migrating chats in Chiapas, Mexico (2018) and Veracruz, Mexico (2014–2015) to determine subspecies and infer breeding location. Endangered chats in British Columbia followed the Pacific Flyway and spent the non-breeding period in Sinaloa and Nayarit, Mexico. Two out of five chats spent the non-breeding period in protected areas, and the most common landcover type used was tropical or subtropical broadleaf deciduous forest. We found no mixing of eastern and western chats in our Mexico sites, suggesting strong migratory connectivity at the subspecies level. Western chats likely originating from multiple breeding latitudes spent the non-breeding period in Nayarit. Eastern Yellow-breasted Chats likely breeding across various latitudes migrated through Veracruz and Chiapas. Our results provide precise migration routes and non-breeding locations, and describe habitat cover types for chats, notably an endangered population in British Columbia, which may be valuable for habitat protection and conservation efforts.

**Supplementary Information:**

The online version contains supplementary material available at 10.1007/s10336-021-01931-8.

## Abstract (de)

**Zug- und Überwinterungsökologie beim Flötenstärling** ***Icteria virens***

Für die meisten Singvogelpopulationen fehlen detaillierte Informationen über Zug und Überwinterung. Hier vergleichen wir die Zug- und Überwinterungslokalitäten von zwei Brutpopulationen des Flötenstärlings *Icteria virens* im südlichen British Columbia, Kanada beziehungsweise in Nordkalifornien, USA. Zwischen 2013–2017 setzen wir in British Columbia Helldunkelgeolokatoren und Satellitensender, in Kalifornien Helldunkelgeolokatoren ein. Mittels einer Kombination aus der Analyse stabiler Wasserstoffisotope mit genetischen Daten aus Federn zogen wir Rückschlüsse auf die Brutorte ziehender Flötenstärlinge in Veracruz und Chiapas, Mexiko sowie überwinternder Flötenstärlinge in Nayarit, Mexiko (2014–2019). Die stark gefährdeten Flötenstärlinge aus British Columbia folgten dem Pazifischen Flyway und überwinterten in Sinaloa und Nayarit, Mexiko. Im Westen auf verschiedenen Breitengraden brütende Flötenstärlinge überwinterten in Nayarit, die im Osten auf verschiedenen Breitengraden brütenden Flötenstärlinge zogen dagegen durch Veracruz und Chiapas. Unsere Ergebnisse liefern genaue Informationen zu Zug und Überwinterung, welche wertvoll für den Erhalt der stark gefährdeten Flötenstärlinge in British Columbia sind.

## Introduction

Most songbird research occurs on the breeding grounds and, therefore, large knowledge gaps exist for migration and non-breeding periods (Marra et al. 2015, 2019). To enact conservation measures to protect and recover songbirds, it is crucial to understand their full annual cycle of breeding, fall migration, non-breeding, and spring migration (Hutto 1989; Faaborg et al. 2010; Marra et al. 2015). Understanding the full annual cycle is important because threats contributing to declining populations may be greatest during migration or the non-breeding period (Rockwell et al. 2017; Wilson et al. 2018). Additionally, events occurring in one phase of the annual cycle may influence events occurring at another phase in some species (carry-over effects, Marra et al. 2015). For example, non-breeding habitat quality can influence body condition (Black-throated Blue Warblers, *Setophaga caerulescens*, Bearhop et al. 2004) and arrival to breeding sites (American Redstarts, *S. ruticilla,* Marra et al. 1998; Norris et al. 2006; Kirtland’s Warbler*, S. kirtlandii*, Rockwell et al. 2012; Cooper et al. 2017), which affects territory access (American Redstarts, Marra et al. 1998). Birds returning early have earlier laying dates, larger clutches, and greater reproductive success (American Redstarts Norris et al. 2004, Reudink et al. 2009; Pied Flycatchers, *Ficedula hypoleuca*, Goodenough et al. 2017, Kirtland's Warbler, Rockwell et al. 2012), although there can be a trade-off in arriving early to the breeding grounds (Brown and Brown 2000).

Identifying the geographic locations of populations throughout the annual cycle is a critical component to evaluate threats and pinpoint drivers of population decline. Additionally, knowing whether breeding populations disperse widely throughout different phases of the annual cycle or are constrained to small geographic areas requires understanding migratory connectivity. Migratory connectivity describes how populations are linked between different phases of the annual cycle through space and time (Boulet and Norris 2006; Bauer et al. 2016). Migrants of populations characterized by strong migratory connectivity remain close (geographically and temporally) through different phases of the annual cycle (Boulet and Norris 2006; Bauer et al. 2016; Cohen et al. 2018). Conversely, migrants of populations characterized by weak migratory connectivity separate (geographically or temporally) during breeding, migration, or non-breeding periods and are more likely to overlap with individuals from other populations (Newton 2006; Bauer et al. 2016; Finch et al. 2017; Cohen et al. 2018). Understanding migratory connectivity helps predict how threats may impact a discrete population and thus provide essential information for recovery planning (Boulet and Norris 2006; Martin et al. 2007; Sheehy et al. 2010; Finch et al. 2017). For example, the loss of critical non-breeding habitat for populations with strong migratory connectivity could extirpate an entire breeding population (Webster et al. 2002). In contrast, for populations with weak migratory connectivity, individuals from one breeding population can use several non-breeding locations, and therefore, the effect of a potential threat may be diffused among multiple breeding populations (Finch et al. 2017; Marra et al. 2019).

Understanding the geographic linkages spanning the full annual cycle is challenging because it is difficult to track small songbirds (Holmes 2007). Band recovery efforts have been ongoing for over 100 years but data are limited and fail to encompass broad geographic areas (Brewer et al. 2006). Fortunately, advances in lightweight technology and computational analyses are continually improving the ways in which we can study birds across their full annual cycle (McKinnon and Love 2018). External trackers such as archival light-level geolocators and GPS tags (McKinnon et al. 2013; Hallworth and Marra 2015), as well as intrinsic markers such as DNA and stable hydrogen isotopes (Hobson and Wassenaar 1997; Lovette et al. 2004; Toews et al. 2017), can be used to better understand breeding, migrating, and non-breeding phases and linking different phases together.

**Fig. 1:**
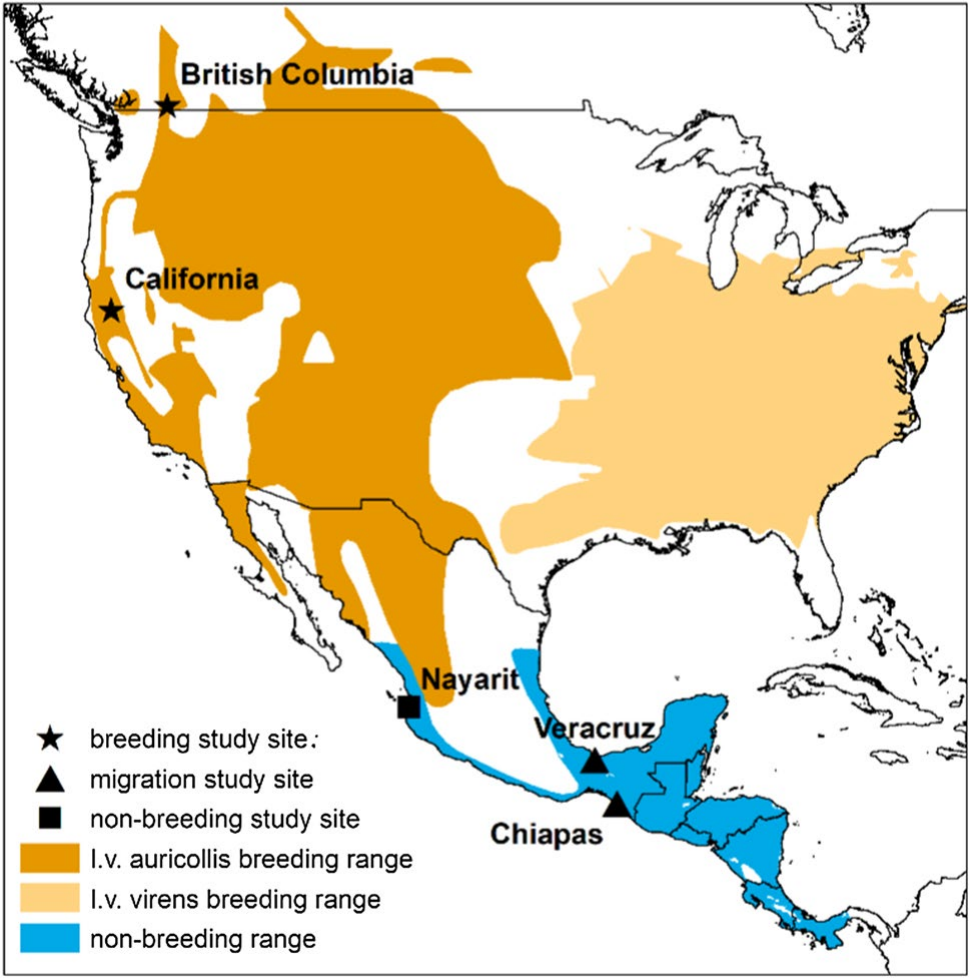
Yellow-breasted Chat (*Icteria virens*) study sites. The eastern (*I.v. virens*) and western *(I.v. auricollis*) subspecies breeding ranges are illustrated along with the species’ non-breeding wintering range (IUCN 2016)

Here, we combine multiple methods to better understand the full annual cycle of a songbird where little is known about the species outside the breeding season. The Yellow-breasted Chat (*Icteria virens*, hereafter, chat) is a Nearctic–Neotropical migrant comprised of two subspecies—eastern (*I.v. virens*) and western (*I.v. auricollis*), which occupy distinct breeding ranges in the United States and southern Canada (Lovette et al. 2004; Smith et al. 2005; Fig. 1). Their non-breeding range spans coastal Mexico and Central America (IUCN 2016). Specific links between breeding and non-breeding populations are not clear (IUCN 2016; Eckerle and Thompson 2020). Throughout their range, chats are at risk of habitat degradation through grazing, succession, and development (Environment and Climate Change Canada 2016, 2019; Eckerle and Thompson 2020). Although globally, the chat is of least concern (Birdlife International 2018), a net loss of ~ four million breeding chats occurred between 1970 and 2017 (Rosenberg et al. 2019; U.S. Geological Survey 2020). Some populations are at risk—in Connecticut and New Jersey, chats are endangered (State of Connecticut 2015; State of New Jersey 2020), and in California and New York, chats are of special concern (Shuford and Gardali 2008; New York State 2019). In Canada, populations of eastern chats occurring in Ontario and populations of western chats occurring in British Columbia (BC) are federally listed as endangered (Government of Canada 2002).

Filling knowledge gaps about migration and non-breeding stages of the annual cycle is a necessary priority for the conservation of chats in BC (Environment and Climate Change Canada 2016). No migration or overwintering information is known (Environment and Climate Change Canada 2016). Working collaboratively with international partners to first identify and then protect habitats throughout their range is desired for the recovery of this population (Environment and Climate Change Canada 2016). Additionally, understanding how this population compares to other chat populations at different parts of the species’ range allows for a deeper contextual basis of full-annual cycle ecology for the species as a whole. Therefore, our study had five main objectives as follows:

1. Describe migration routes and non-breeding locations for chats breeding in BC using archival GPS tags and light-level geolocators.
2. Examine whether non-breeding locations are in protected areas and characterize land cover within 1 km.
3. Describe non-breeding locations for chats breeding in California, by using light-level geolocators.
4. Determine whether chats sampled outside the breeding season in Nayarit, Veracruz, and Chiapas, Mexico are of the eastern or western subspecies.
5. Infer breeding origins for chats sampled during the non-breeding season in Nayarit, Mexico, and during the migration period in Veracruz and Chiapas, Mexico.

## Materials and methods

We studied chats in five study areas at different phases of the annual life cycle (Fig. 1). Breeding chats were studied in southern BC (49.182° N, 119.550° W) and northern California (40.718° N, 122.838° W) in dense, shrubby areas near riparian habitats (Eckerle and Thompson 2020). Study sites in BC were adjacent to the channelized south Okanagan River (McKibbin and Bishop 2010). In California, sites were located along the Trinity River in the 64 km between the Lewiston Dam and the confluence with the North Fork Trinity (Rockwell and Stephens 2018). Migrating chats were studied in Veracruz (17.989° N, 95.511° W) and Chiapas (15.552° N, 93.205° W). The Veracruz study site was located at the northern end of the Isthmus of Tehuantepec and consists of a marshy area with a mix of shrubs and trees < 5 m tall. The Chiapas site was located within the northern end of the La Encrucijada Biosphere Reserve on the Pacific coast (Gahbauer et al. 2016). The Chiapas site consisted of lagoons and wetlands with interspersed shrubs and grasses. Non-breeding chats were studied near the town of San Francisco, Nayarit (20.908° N, 105.398° W) where the habitat consisted of tropical semi-deciduous forests and edge habitats near riparian areas.

### Bird captures and handling

Chats were captured in mist-nets passively and using call playback, often paired with a decoy. We primarily tracked males because females are cryptic and more difficult to resight and recapture compared to males. Birds were aged and sexed following criteria in Pyle (1997) and banded with numbered aluminum bands. Our ability to age and sex birds varied depending on the time of year, but age categories included: hatch-year, second year, after second year, and after hatch-year (Pyle 1997). In BC, California, and Nayarit, birds were also given three colour bands to aid in resighting. One right outer rectrix (R6) was collected for genetic analyses (and corticosterone, Mancuso et al. 2021, pending). One right inner secondary feather (S4–S6) was collected for hydrogen stable isotope analyses, except in California where no secondaries were collected, but instead, two outer rectrices were taken. Feathers were collected from breeding chats in 2018 in BC and California.

In Veracruz, feathers were collected from chats in March, May, August–November 2014, and March 2015. In Chiapas, feathers were collected during September–October 2018. We assumed that birds sampled in Veracruz and Chiapas were all migrating based on date. In Nayarit, feathers were collected from chats in January–February 2017–2019 and we assumed that the sampled chats were at their non-breeding sites based on date.

At breeding sites, tracking devices were attached to the backs of birds by using a leg-loop harness (Rappole and Tipton 1991) with Stretch Magic™ string. Harness size and fit were based on mass equations from Naef-Daenzer (2007). Chats have high site fidelity to breeding areas, facilitating re-sighting and recapturing efforts to retrieve tracking devices in subsequent years (McKibbin and Bishop 2012).

We deployed 14 archival 1.0-g GPS tags (Lotek Wireless Inc. 2020) on adult male chats breeding in BC to identify migration routes, stopover sites, and non-breeding locations. Seven Lotek model GPS-10 tags deployed in 2016 could acquire up to 10 fixes, which we scheduled for 10–30 days apart. Seven Lotek model GPS-10 Swift tags deployed in 2017 could acquire up to 80 fixes and were scheduled to obtain fixes every 4 days. Tags were scheduled to take the fix at 16:00 or 17:00 GMT (9:00 or 10:00 PDT) where birds should be awake and active. We tested the same type of GPS tags for another study before deployment at a stationary location in BC and found that accuracy was within a few meters for many points, and 90% of points were within 100 m (Mancuso et al. 2021). According to the manufacturer, the lowest levels of accuracy (which ought to be removed) are 300 m (Lotek Wireless Inc. 2018). We deployed 37 Biotrack archival light-level geolocators on chats in BC during 2013–2014, and 51 geolocators on chats in California during 2014–2016. We tagged mostly adult males, but also three hatch-year chats of unknown sex, and one adult female.

To ensure that geolocators and GPS tags did not reduce the survival of chats, we compared the apparent annual survivorship of birds with tracking devices to birds banded, but not given tracking devices. The dataset spanned 2013–2019 for adult birds in BC where field technicians resighted chats in known breeding territories. Previously identified breeding territories were visited from dawn until ~ 1100 from May to July and if occupied, the unique colour-band combination of the male chat was determined using binoculars and high-zoom cameras. Call-playback surveys (consisting of 10 min of silence, 30 s play-back, 2 min silence, 30 s play-back, and 2 min silence) were used to determine if a territory was occupied if it was not already obvious upon arriving at the territory. Apparent annual survivorship was calculated using the RMark package (*v.2.2.7*; Laake 2013 within *v 3.5.1;* R Core Team 2018) using Cormack-Jolly-Seber models (Cormack 1964; Schwarz 2001). The parameters included intercepts for apparent annual survivorship (ϕ) and detection probability (p). This model was previously determined to be the best model for the chat population in our BC study area (McKibbin and Bishop 2012).

### Determining migration and non-breeding sites from breeding chats

To determine non-breeding sites of chats, geolocators were analyzed using the Geolight package (*v2.0.1;* Lisovski and Hahn 2012) in R following methods by Lisovski et al. (2020). We determined twilights using the preprocessLight function from the TwGeos package (*v0.1.2;* Lisovski et al. 2016) with a light threshold value of 0.5. To remove unlikely Twilight estimates, we used the twilightEdit function from the TwGeos package (window set to 4, outlier minutes set to 45, and stationary minutes set to 25). We constrained non-breeding dates to Nov. 15–Mar. 1 to avoid location estimates during the spring equinox, a time where equal day and night lengths make it notoriously difficult to determine latitude (Ekstrom 2004) We used the findHEZenith function from the TwGeos package to determine the most likely sun elevation angle from Nov. 15 Mar. 1 (Hill and Braun 2001; Ekstrom 2004). Twilights were converted to latitude and longitude estimates using the coord function in Geolight*.* Geolocators generally have much higher error estimates for latitude than longitude, especially in forested habitats (Fudickar et al. 2012; Biotrack Limited 2013). Therefore, we present summary statistics on the longitude values for each geolocator.

Latitude and longitude data were downloaded directly from GPS tags retrieved from chats. We removed points considered to have low accuracy (dilution of precision values greater than 20, Lotek Wireless Inc. 2018). Stopover sites were defined as any location with consecutive fixes during migration (either 4 days or 10–15 days apart). Approximate migration routes were created by connecting GPS points with lines in ArcMap 10.7.1 (ESRI 2019).

We examined the protected areas of Mexico (Comisión Nacional de Áreas Naturales Protegidas 2020a, b; UNEP-WCMC 2020) to assess whether chats spent the non-breeding period in protected areas. These mapped polygons conform to the definition of protected areas from the International Union for the Conservation of Nature or the Convention on Biological Diversity, which generally includes geographic areas that are managed for the conservation of nature (UNEP-WCMC 2017). To visualize habitat, we mapped satellite imagery within a 1 km radius of the non-breeding location for each chat. To understand land over at non-breeding sites, we summarized the percent of each land cover category within these areas using raster data from the 2015 Land Cover of North America at 30 m (Commission for Environmental Cooperation 2020).

### Determining chat subspecies

We used genetics and stable hydrogen isotopes to determine the breeding origin from chats sampled in their overwintering and migration sites. Previous genetic analyses based on the ATPase gene in the mitochondrial genome identified 18 unique haplotypes within 34 chats sampled from across their North American distribution, including both subspecies (Lovette et al. 2004). Importantly, the authors identified several single nucleotide polymorphisms (SNPs) diagnostic of western and eastern chats. Here, we designed and validated novel TaqMan® genotyping assays targeting diagnostic SNPs in the mitochondrial ATPase gene to identify eastern and western chats, via two fluorescent probes in each reaction. We designed two genotyping assays (Iv_ATPase_SNP200; Iv_ATPase_SNP778) using the Custom TaqMan® Assay Design Tool provided by Life Technologies. We validated the assays by genotyping 79 reference samples of known locality and subspecies origin across the North American range of *I. virens* as part of the Bird Genoscape Project (www.birdgenoscape.org): 39 samples of *I.v. virens* (Indiana, Kentucky, Alabama, Missouri) and 40 samples of *I.v auricollis* (Montana, California, Oregon, BC). We then used the Taqman® genotyping assays to identify the subspecies of 211 samples, including 38 from Chiapas, 19 from Nayarit, and 154 from Veracruz. Detailed descriptions of our DNA extraction and genotyping methods can be found in Online Resource 1.

### Breeding origin from non-breeding and migrating chats

Hydrogen isotope ratios in precipitation (*δ*^2^H_p_) vary latitudinally and ratios are more depleted at northern latitudes (more negative) and less depleted towards the equator (less negative). Hydrogen isotope ratio signatures within animal tissues reflect where the animal tissue was grown (Hobson and Wassenaar 1997; Hobson et al. 2012). In combination with genetics, feather stable hydrogen isotope values (*δ*^2^H_f_) were used to infer breeding location from chats sampled on their non-breeding grounds in Nayarit (*n* = 40) and migrating chats in Veracruz *(n* = 125) and Chiapas (*n* = 45, Table 2). The isotopic signature of feathers represents conditions in the breeding grounds, as chats complete their pre-basic moult before fall migration (Pyle 1997, pers. obs). Feathers of known origin for calibration purposes were collected from breeding chats in BC (*n* = 15) and California (*n* = 15). Ten chat additional feathers were provided by Klamath Bird Observatory from two breeding sites in Oregon from 2018 for calibration. One inner secondary feather was used for stable hydrogen isotope analyses, except for Oregon chats where one rectrix was used instead.

The feathers were prepared following Bontempo et al. (2014). Each feather was washed three times in a 2:1 solution of diethyl ether: methanol by sonicating for 1 min. The feathers were air-dried in a fume hood overnight. The rachis was removed, and the remaining vane was placed in a glass scintillation vial and then cut into the finest powder possible using stainless-steel scissors.

Stable hydrogen isotope analyses were performed by the Laboratory of Stable Isotope Science at the University of Western Ontario, Canada. Feathers were analyzed using a Thermo Scientific High-Temperature Conversion Elemental Analyzer (TC/EA) plus a continuous flow Thermo Scientific Delta V Plus isotope ratio mass spectrometer. Samples and standards were held at room temperature for > 72 h to allow for hydrogen isotope equilibration. Approximately 0.3–0.4 mg of sample were loaded into silver capsules, and then into a Zero Bank Autosampler. Each sample was purged using dry helium at 90 ml per minute for 7 min to remove any moisture. The samples were then dropped automatically into the TC/EA reactor column. The internal design of the TC/EA follows the design of Dr. Christine France at the Museum Conservation Institute, Smithsonian Institute, whereby chromium powder (99.5% purity, 100 mesh size), quartz chips, and quartz wool spacers are added to achieve the correct height. The TC/EA allows samples to undergo pyrolysis by heating to 1120 °C. Gas separation occurred in a gas chromatography column heated to 90 °C and packed with a 0.6 M molecular sieve.

The hydrogen isotope compositions were calibrated using two standard reference materials: (1) Caribou Hoof Standard, and (2) Kudu Horn Standard based on the updated values from Soto et al. (2017). Keratin powder was used for monitoring drift and correction. Final hydrogen isotope ratios in feathers (*δ*^2^H_f_) are presented in parts per thousand (‰) normalized to Vienna Standard Mean Ocean Water.

We used the package IsoriX (*v0.8.2*; Courtiol et al. 2019) in R to create a North American precipitation isoscape (Courtiol and Rousset 2017). This precipitation isoscape was calibrated to reflect *δ*^2^H_f_ and was used to predict the breeding location for chats sampled during the migration or the non-breeding period. To create the precipitation isoscape, *δ*^2^H_p_ values from Canada, the USA, and Mexico were downloaded on December 7, 2019, from the Global Network of Isotopes in Precipitation (IAEA/WMO 2019). The dataset included growing season monthly values (> 0 °C) for each station between 1953 and 2019. The precipitation isoscape was created using the isofit function in IsoriX. This precipitation isoscape was calibrated to reflect *δ*^2^H_f_ values of chats by using 47 samples of known origin location, with 38 prepared ourselves plus 9 rectrix samples from Hobson et al. (2012, Online Resource 2). Calibration was completed using the calibfit function within IsoriX, which fits a linear mixed-effects model that accounts for the uncertainty associated with making predictions from isoscapes by factoring in the variance and covariance associated with predictions across geographic space (Courtiol et al. 2019).

To infer the geographic location of chats using stable hydrogen isotope ratios in feathers, samples of unknown origin were divided into 10‰ bins spanning the data range, which resulted in nine bins. The purpose of the bins was to group individuals with similar isotopic signatures together for summarizing the area of origin using the isofind function of IsoriX. Based on our genetic results, we used the mask option of IsoriX to constrain the geographic assignment areas of chats sampled in Veracruz and Chiapas to the range of the eastern subspecies and chats sampled in Nayarit to the range of the western subspecies (IUCN 2016).

## Results

**Fig. 2:**
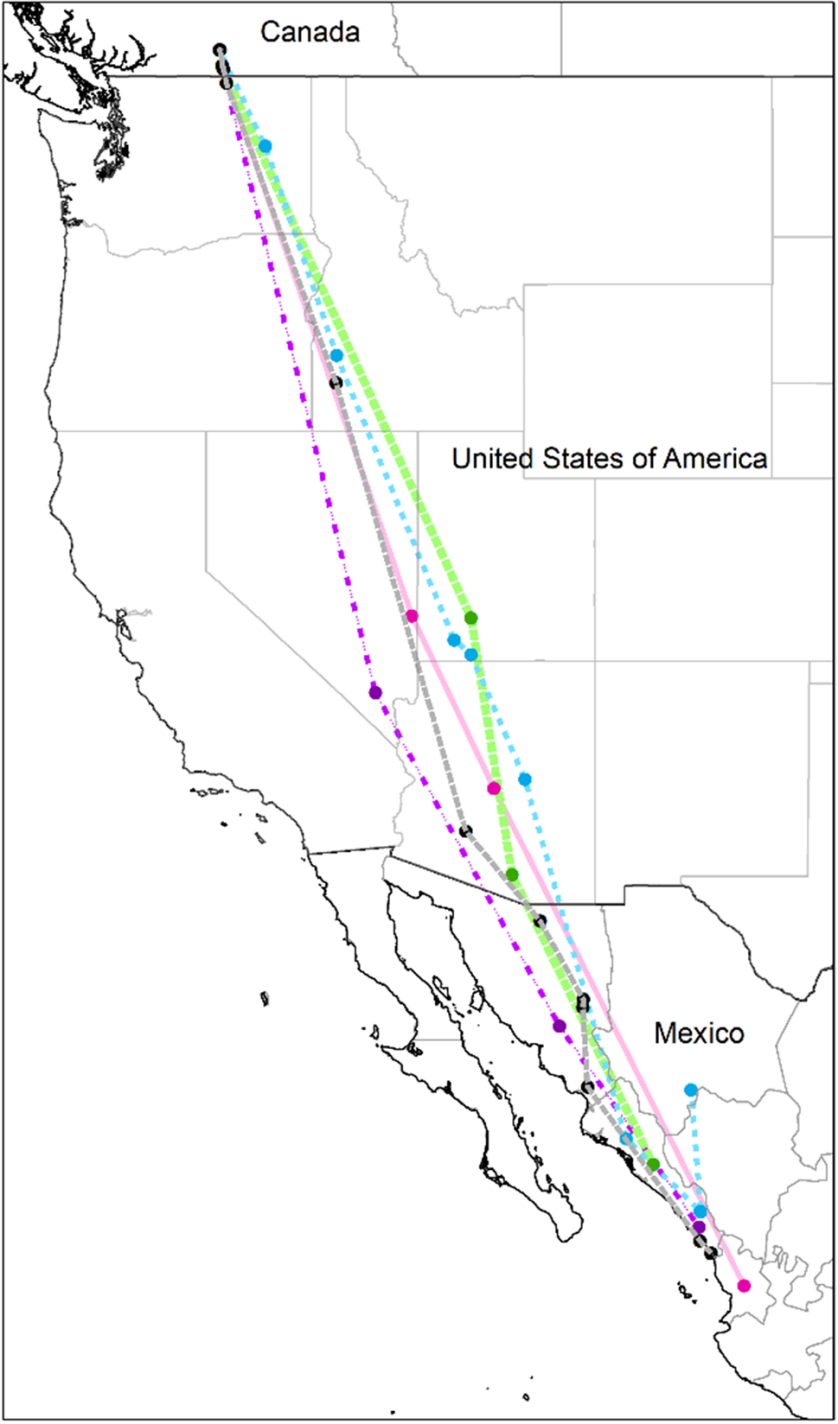
Estimated fall migration routes of western Yellow-breasted Chats (*Icteria virens*). Five male chats were tracked using Lotek PinPoint GPS units from breeding sites in the South Okanagan Valley of British Columbia, Canada to non-breeding sites in Sinaloa and Nayarit, Mexico. GPS units were deployed in 2016 and 2017. GPS fixes were connected with straight lines to approximate migration route

Six of 14 (42.9%) GPS tags were retrieved from chats in BC. Five tags had between four and 52 data points, but the other tag failed to collect data. Chats breeding in BC migrated south via the Pacific Flyway in early September through Washington, Oregon, Idaho, Nevada, Utah, and Arizona, then Sonora, Chihuahua, Sinaloa, and Nayarit (Fig. 2). We identified one stopover site in Idaho, where a chat remained for a minimum of 8 days between September 14 and 22, 2017. The stopover site was a narrow riparian strip surrounded by a human-modified landscape (Online Resource 3).

**Fig. 3:**
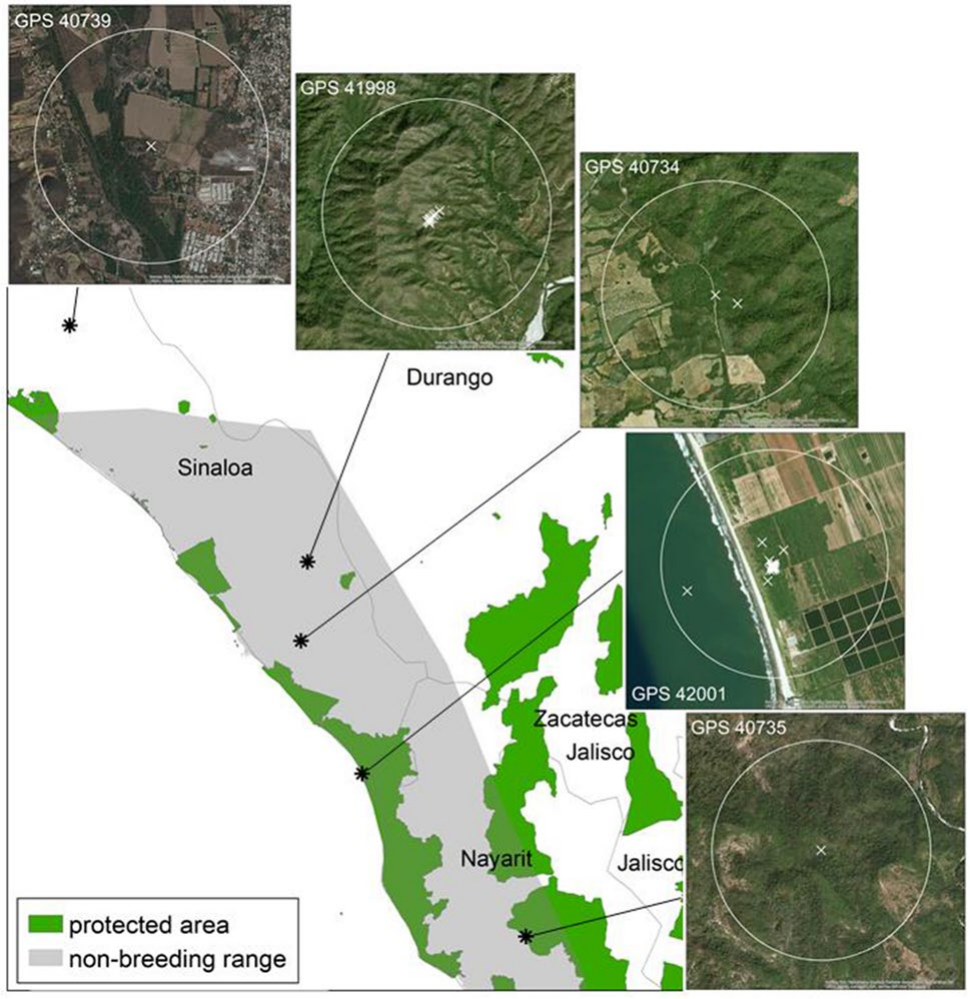
Non-breeding areas of western Yellow-breasted Chats (*Icteria virens auricollis*) that bred in British Columbia. Asterisks denote non-breeding locations as determined from tracking chats with GPS tags deployed in 2016 and 2017. The square is our Nayarit study site indicated in Fig. 1. Satellite imagery of non-breeding locations provided for visualizing habitat. Protected areas of Mexico were from the United Nations Environment World Conservation Monitoring Centre (UnEP-WCMC 2020). Non-breeding range provided by IUCN (2016)

Chats arrived at their non-breeding grounds in Sinaloa or Nayarit in October. Two chats spent the non-period in protected areas; one in the Biosphere Reserve Marismas Nacionales Nayarit and one in a natural resource protected area, Cuenca Alimentadora del Distrito Nacional de Riego 043 Estado de Nayarit (Comisión Nacional de Áreas Naturales Protegidas 2020a, b; UNEP-WCMC 2020). Satellite imagery of non-breeding locations showed that chats resided in a mix of natural and human-modified landscapes (Fig. 3). The major land cover type within 1 km of non-breeding sites for three of five chats was tropical or sub-tropical broadleaf deciduous forest (Table 1). The major land cover type within 1 km of non-breeding sites for the other 2 chats was cropland (Table 1).

**Table 1:** Mexico land cover analyses within one kilometer of western Yellow-breasted Chat (*Icteria virens auricollis*) non-breeding locations as determined by GPS tags

Land Cover Category (%)	GPS 40,739	GPS 40,735*	GPS 41,998	GPS 40,734	GPS 42,001*
Tropical or sub-tropical broadleaf deciduous forest	4.73	96.02	96.39	71.25	–
Tropical or sub-tropical grassland	–	1.58	–	0.17	–
Temperate or sub-polar grassland	–	0.14	–	–	
Cropland	79.01	2.26	1.49	28.58	62.65
Barren lands	0.72	–	–	–	2.58
Urban	13.65	–	2.12	–	–
Water	1.89	–	–	–	–
Null	-–	–	–	–	34.78

Dashes indicate 0%. Asterisks denote that this chat spent the non-breeding season in a protected area

We retrieved 17 of 37 (45.9%) geolocators from chats in BC and 9 of 51 (17.6%) from chats in California. Of these, six from BC and six from California contained usable light data. Longitudinal estimates suggest chats breeding in California spent the non-breeding season in western Mexico, except for one tag where the average longitude was over the Pacific Ocean (Table 2). Longitudinal estimates suggest chats breeding in BC spent the non-breeding season in western or central Mexico (Table 2).

**Table 2:** Longitude estimates during the non-breeding period (Nov 15–March 1) from light-level geolocators deployed on Yellow-breasted Chats (*Icteria virens*)

Bird ID	Mean Longitude	Median Longitude	Standard Error	*N*
California, USA				
GXLR	− 126.3	− 130.1	23.3	18
LRLX	− 107.6	− 107.6	3.6	185
RXLD	− 107.6	− 107.8	4.1	97
WWLX	− 107.5	− 107.5	1.6	213
WXLR	− 107.1	− 108.3	6.5	28
LXLG	− 104.0	− 104.0	3.3	135
British Columbia, Canada				
DXOD	− 106.6	− 108.0	6.9	31
YXYR	− 106.6	− 106.8	4.5	126
XGRL	− 105.0	− 105.3	7.4	21
YXYY	− 104.6	− 104.4	4.3	119
GXYG	− 101.5	− 102.0	5.7	52
XGYY	− 98.8	− 99.1	5.2	79

We did not find any evidence of tracking devices resulting in reduced survivorship of chats. The apparent annual survivorship (± standard error) of chats with tracking devices in BC (which were primarily males) between 2013 and 2019 was 0.62 ± 0.05 (*n* = 50). The apparent annual survivorship of birds without tracking devices was 0.55 ± 0.04 (*n* = 137).

### Genetic separation of eastern and western subspecies

Seventy-six of 79 reference samples collected from the chats on the breeding grounds were successfully genotyped at both newly developed TaqMan® SNP assays (Iv_ATPase_SNP200; Iv_ATPase_SNP778); two of the remaining individuals were successfully genotyped at one assay, while one sample from California did not amplify. All genotyped reference individuals were accurately assigned to their geographic subspecies of origin (Online Resources 1). All samples from Nayarit were genotyped at both SNP assays and assigned to the western *I.v. auricollis*. All samples from Chiapas were genotyped at both SNP assays and assigned to the eastern *I.v. virens*. For Veracruz, 143 of 154 samples were successfully genotyped (all but three at both assays); all were assigned to the eastern *I.v. virens* (Online Resource 1). Our Nayarit non-breeding site with western chats is near other western chat non-breeding sites reported by Lovette et al. (2004). Our Chiapas and Veracruz migration sites with eastern chats were near eastern non-breeding sites reported by Lovette et al. (2004); however, our research expands the knowledge of which chat subspecies is present along the Pacific coast of Mexico.

### Inferred breeding origin from non-breeding and migrating chats

**Fig. 4:**
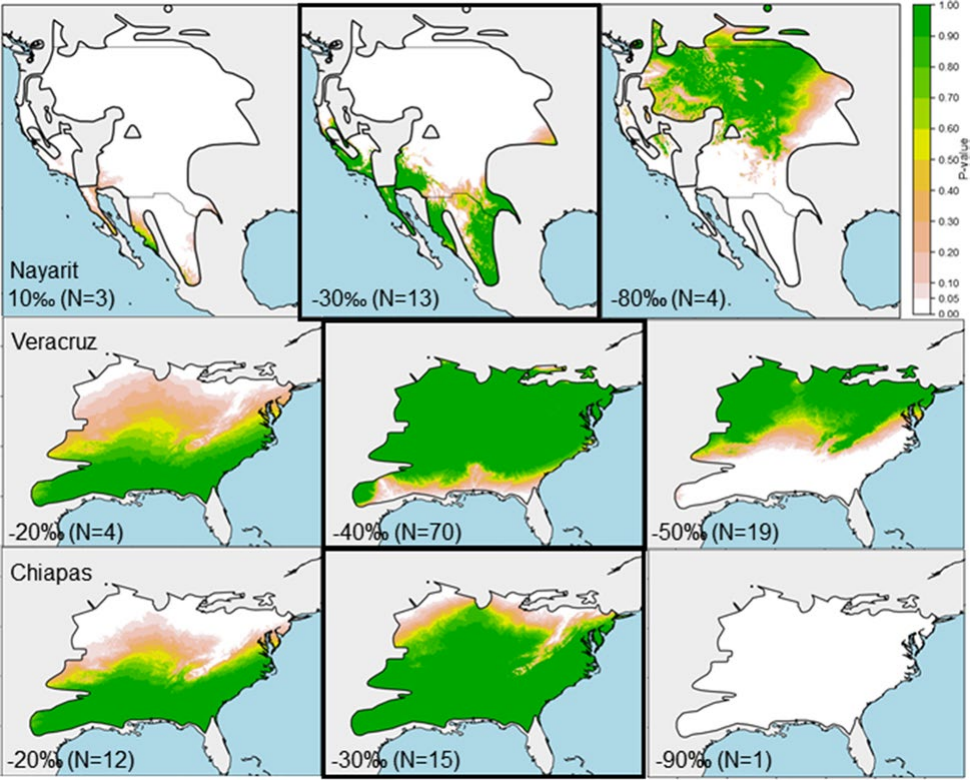
Yellow-breasted Chat breeding origins inferred from stable hydrogen isotope ratios in feather samples collected outside the breeding season. Stable hydrogen isotope values were divided into bins spanning 10‰—i.e. the − 20‰ bin corresponds to hydrogen isotope values of − 20 < − 30‰. Group assignment location estimates are shown for the least depleted bin which corresponds to the most southerly geographic estimates (left), the bin containing the greatest number of birds (center, outlined in black), and the most depleted bin corresponding to the most northerly geographic estimates (right). Chats sampled in Nayarit were of the western subspecies and, therefore, constrained to the western chat breeding range (IUCN 2016). Chats sampled in Veracruz and Chiapas were of the eastern subspecies and therefore constrained to the eastern chat breeding range (IUCN 2106). The greenest areas and larger *P* values correspond to higher assignment probabilities

Stable hydrogen isotope values spanned a large range of values, indicating that birds likely originated from various latitudes across the breeding range. The *δ*^2^H_f_ values for chats sampled in Nayarit ranged between − 87.4‰ and − 10.7‰. Most (32.5%) samples fell within the − 30 to − 40‰ bin, which corresponds to assignment origins in the southern and western portion of the *I.v. auricollis* breeding range (Table 3, Fig. 4). The *δ*^2^H_f_ values for chats sampled in Chiapas ranged between − 90.4‰ and − 20.1‰. Most (33.3%) samples fell within the − 30 to − 40‰, which corresponds to birds originating from the central portion of the *I.v. virens* breeding range. The *δ*^2^H_f_ values for chats sampled in Veracruz ranged between − 58.2 and − 25.5‰. Most (56.0%) samples fell within the − 40 to − 50‰ bin, corresponding to assignment origins in the south-central portion of the *I.v. virens* breeding range.

**Table 3:** Feather stable hydrogen isotope ratio (δ^2^H_f_) summary

δ^2^H_f_ (‰) Range	Nayarit (*auricollis*)			Chiapas (*virens*)			Veracruz (*virens)*		
	Mean ± SE	*N*	%	Mean ± SE	*N*	%	Mean ± SE	*N*	%
− 10 > − 20	− 15.4 ± 1.4	3	7.5	–	–		–	–	–
− 20 > − 30	− 28.0 ± 0.5	2	5.0	− 27.1 ± 0.9	12	26.7	− 28.0 ± 0.9	4	3.2
− 30 > − 40	− 35.8 ± 0.2	13	32.5	− 34.6 ± 1.0	15	33.3	− 36.4 ± 0.5	32	25.6
− 40 > − 50	− 45.0 ± 0.2	11	27.5	− 43.0 ± 0.4	10	22.2	− 44.1 ± 0.3	70	56.0
− 50 > − 60	− 52.9 ± 0.3	3	7.5	− 52.9 ± 1.2	6	13.3	− 52.9 ± 0.5	19	15.2
− 60 > − 70	− 61.58 ± 0.6	3	7.5	–	–	–	–	–	–
− 70 > − 80	− 72.5	1	2.5	− 71.4	1	2.2	–	–	–
− 80 > − 90	− 84.8 ± 0.7	4	10	–	–	–	–	–	–
− 90 > − 100	–	–	–	− 90.4	1	2.2	–	–	–
Total	− 45.4 ± 0.5	40	100	− 39.1 ± 1.9	45	100	− 43.0 ± 0.6	125	100

Feathers were collected from Yellow-breasted Chats (*Icteria virens*) of both subspecies from 3 different sites in Mexico between 2014 and 2019. Nayarit chats were sampled during the non-breeding period in January, and February. Chiapas and Veracruz chats were sampled during migration in March, May, August, September, October, and November. Stable hydrogen isotope ratios are reported per thousand (‰) standardized to Vienna Standard Mean Ocean Water. The most depleted stable hydrogen isotope values (more negative) correspond to northerly latitudes and the least depleted stable hydrogen isotope values (less negative) correspond to southerly latitudes. Dashes indicate no data

## Discussion

We have described in detail the migration and non-wintering ecology of an endangered population of chats that breeds in BC, as well as contributed to a broader understanding of migration ecology for the species. We found that endangered BC chats migrated via the Pacific Flyway towards non-breeding sites Sinaloa or Nayarit. BC chats that spent the non-breeding season in western Mexico share this region with other western chats originating from multiple breeding latitudes, likely including those that bred in California. Although our sample size is small, two out of five chats tracked overwintered within protected areas. Protecting additional sites in western Mexico that support patches of ongoing early-successional habitat and tropical or subtropical broadleaf deciduous forest will be beneficial to conserving western chats generally, and specifically, the endangered population that breeds in BC.

In Chiapas and Veracruz, we found that migrating chats were of the eastern subspecies. Based on isotopes, it appears that eastern chats originating from multiple breeding latitudes use these migration sites. However, more detailed information on non-breeding site use would be valuable to complete our understanding of the full annual cycle for this subspecies.

### Subspecies separation

Our method of SNP genotyping using mitochondrial DNA to determine eastern and western subspecies was novel and successful. We found no co-occurrence of the eastern and western subspecies in Mexico, as previously found in Lovette et al. (2004) which suggests strong connectivity at the subspecies scale. Since we found only western chats in Nayarit, when we combine our results with those of Lovette et al. (2004), it appears that the separation between the eastern and western subspecies in Mexico likely occurs between coastal Chiapas and Oaxaca. If this is true, the western subspecies may have a smaller non-breeding range restricted to western Mexico, compared to the eastern subspecies that go as far south as Central America. However, more sampling in sites farther south would be necessary to confirm this idea.

### Migration ecology

The Pacific Flyway that western BC chats used is common for songbirds breeding west of the Rocky Mountains (Hutto 1989; Brewer et al. 2006). The timing and general route of the fall migration path for the western BC chat were similar across all five individuals, suggesting strong connectivity during this migration period for this population, although sample size is limited. Therefore, this population may be at risk of threats along the migratory route, such as wildfires. We detected one stopover site in Idaho, which consisted of a small strip of riparian habitat located within a heavily modified landscape, highlighting that even small areas of suitable habitat can be used by migrating chats.

Eastern chats migrating through Veracruz and Chiapas likely originated from various latitudes throughout their breeding range. The larger span in hydrogen isotope ratio of Chiapas chats suggests that chats migrating through Chiapas may originate from a larger geographic range than chats migrating through Veracruz. Banding recovery data link the individuals in the northeastern portion of the breeding range to Veracruz (Mancuso 2020), suggesting that our isotope assignment surfaces may be accurate, although feathers from re-encounters in Mexico would be valuable for validation. Because we found exclusively eastern chats in Veracruz and Chiapas, it is likely that the eastern subspecies migrates via the Isthmus of Tehuantepec, a common migratory route for birds between the Gulf of Mexico and the Pacific coast of Mexico (Howell and Webb 1995; McAndrews and Montejo Dıaz 2010; Kochert et al. 2011; Cabrera-Cruz et al. 2017).

### Non-breeding ecology

The five breeding chats tagged with GPS tags spent the non-breed period within a few hundred kilometers of each other, suggesting some degree of geographic spread but overall on the continental scale, relatively strong migratory connectivity for this population. However, there is likely overlap with other breeding populations in Nayarit based on our stable isotope analyses and potentially the geolocator results, suggesting that if one considers non-breeding chats near Nayarit as a larger population, they are exhibiting weak migratory connectivity. This highlights the challenges of classifying the degree of migratory connectivity exhibited within populations. The use of GPS tags on chats in more populations would allow for a quantification of the strength of migratory connectivity and a better understanding of movement dynamics (Mantel 1967; Ambrosini et al. 2009; Cohen et al. 2018).

Chats with GPS tags that bred in the south Okanagan Valley spent the non-breeding period in Nayarit and Sinaloa, Mexico. Satellite imagery showed BC chats used a range of natural and human-modified habitats during the non-breeding period. The most commonly used land cover type within 1 km of non-breeding sites was tropical or sub-tropical broadleaf deciduous forest followed by cropland. Previous documentation of chats in their non-breeding range in Mexico suggests that they prefer secondary growth forest with canopy heights less than 10 m (Hutto 1989; Rappole et al. 1998). One chat spent the non-breeding period in the Biosphere Reserve Marismas Nacionales Nayarit, which is a 2000 km^2^ wetland area of international importance designated in 1995 (Comisión Nacional de Áreas Naturales Protegidas 2020b; UNEP-WCMC 2020). However, aerial imagery and land cover data showed that this site is dominated by agriculture, which is counter-intuitive based on the definition that these sites are regulated to protect nature. Likewise, our Chiapas site was located within the La Encrucijada Biosphere Reserve yet habitat modification, cattle grazing, and pesticide use were present. A better understanding of regulations and enforcements in place to conserve nature in Mexican protected areas would be valuable to assess where improvements can occur if necessary.

A second chat spent the non-breeding period in a natural resource protected area identified as Cuenca Alimentadora del Distrito Nacional de Riego 043 Estado de Nayarit which is a conglomerate of smaller sites encompassing 23,290 km^2^ designated in 2002 (Comisión Nacional de Áreas Naturales Protegidas 2020b; UNEP-WCMC 2020). The site is dominated by tropical or sub-tropical broadleaf deciduous forest. Our study site in Nayarit consisted of a mix of natural areas, agriculture, and cattle grazing, but within these areas, chats were found in the dense, shrubby vegetated areas – further supporting the idea that chats can use landscapes that have some degree of human modification. During banding in Nayarit, we found that 8.5% of chats returned to the same Nayarit site in subsequent non-breeding seasons, indicating some degree of non-breeding site fidelity (Mancuso 2020).

With the main threat to chats being loss of habitat (Environment and Climate Change Canada 2016; Eckerle and Thompson 2020), the expansion or addition of new protected areas that offer suitable ongoing early successional habitat in Nayarit and Sinaloa would help protect this population, plus many other neotropical endemic and migrant bird species that spend the non-breeding season in western Mexico (Palomera-García 1994; Castellón et al. 2017). Outside protected areas (and potentially even within protected areas), chat habitat in western Mexico may be at risk of habitat loss, fragmentation, or degradation due to conversion for agriculture, grazing, and human development (Challenger 2019; Valdez 2019). Tropical and subtropical deciduous forests along the Pacific coast of Mexico are hotspots of biodiversity and are at high risk of habitat loss due to land conversion and a high human footprint (Venter et al. 2016; Wilson et al. 2019).

### Survivorship

It is encouraging that tracking devices did not affect the apparent annual survivorship for chats. These results agree with other reviews that found tracking devices do not negatively affect survivorship for most songbirds (Bridge et al. 2013; Streby et al. 2015), except highly aerial species (Morganti et al. 2017). Survivorship estimates of 0.62 for birds with tracking devices are similar to the survivorship estimate of 0.65 for males in the study area from 2001 to 2006 (McKibbin and Bishop 2012) and for adult chats within the Northern Rockies bird conservation region of 0.61 ± 0.04 between 1992 and 2006 (DeSante et al. 2015). These values are higher than the survivorship estimate of 0.55 for chats without tracking devices, but this low estimate likely reflects reduced resighting effort (Martin et al. 2017); we prioritized fieldwork on territories of chats with devices.

## Limitations

One challenge of using *δ*^2^H_p_ is that assignments can only be made to large geographic areas because the large variation of *δ*^2^H_f_ (and underlying *δ*^2^H_p_) make it difficult to determine with certainty the origin of unknown samples. The inter-annual variation of *δ*^2^H_p_ in North America is such that the accuracy of assigning an unknown sample based on *δ*^2^H_p_ cannot confidently be less than 12° of latitude (Farmer et al. 2008). Therefore, the range of the western chats is more suitable for this type of analysis due to their range spanning greater changes in latitude than the eastern chat. Our use of 10‰ bins for analyses was to create group assignments for samples with similar *δ*^2^H_f_ signatures, but chats in adjacent different bins did not necessarily come from different geographic areas, as can be seen from the high variation in *δ*^2^H_f_ values in our known, reference samples (Online Resource 2). Showing the geographic assignments visually from our most depleted to our least depleted bins allows for a better understanding of the geographic separation between assignment locations, i.e. Chiapas birds in − 20‰ > − 30‰ definitely came from different latitudes than birds in the Chiapas − 80‰ > − 90‰ bin because there is no geographic overlap (Fig. 4). Our calibration model could be further improved by the addition of more chat feather samples from known breeding sites, especially in the eastern portion of their range where samples were lacking. This is obvious for depleted values of eastern chats, as our model did not have any overlap of the range with *δ*^2^H_f_ signatures between − 90‰ > − 100‰ (suggesting birds came from more north than their mapped range).

Our sample size of results from GPS-tagged chats was small (*N* = 5) and so our inferences about migration routes, stopovers, and overwintering habitat are limited. It would be valuable to deploy more GPS tags on birds in the endangered BC population and other populations across the chat range to identify additional migration routes, stopover sites, and non-breeding locations. We tracked males, and it would be of high conservation value to see whether any sex-based differences exist in non-breeding areas for chats breeding in BC, as habitat segregation between sexes has been identified on the non-breeding grounds for other songbirds (Lynch et al. 1985; Parrish and Sherry 1994).

Unfortunately, the use of Lotek geolocators failed to collect adequate light data needed to identify precise non-breeding locations of chats that had bred in California and BC. Substantial shading of our geolocators might be attributed to the chat’s preference for dense, shrubby vegetation (Eckerle and Thompson 2020). Another possibility is the sensor may have been obscured by interscapular feathers, despite the light sensor being elevated on a short stalk (Mancuso pers. obs). The exact causes of low-quality data are unclear, but similar issues with geolocators were encountered with Gray Catbirds (*Dumetella carolinensis*) tagged in the same BC study area (Mancuso 2020). Large errors in latitude as high as 365 km have been documented elsewhere in tropical forested habitats (McKinnon et al. 2013). In contrast, geolocators worked well in the same BC site for Veeries (*Catharus fuscescens*), although they used a combination of Lotek and Migrate Technology geolocators (Kardynal and Hobson 2017).

Our ability to detect stopovers was severely limited by the time between GPS fixes being 4 days apart in the best-case scenario. Little is known about the stopover ecology of chats, but in Texas, the average length of stay during fall migration was 4 days based on 3 chats, and 9 days for spring migration based on 1 chat (Rappole and Warner 1976). Therefore, more stopovers were likely used that we were unable to detect.

## Conclusions

We have filled knowledge gaps about migration and non-breeding stages of the Yellow-breasted Chat, notably the endangered BC population. While working collectively with international partners, we have identified with high precision, migration routes, one stopover site, and non-breeding locations. We have determined two non-breeding locations that are already protected but may still have room for improvement and identified three sites that are currently not protected that may benefit from the creation of future protected areas. Using land cover data, we have found that chats tend to use tropical or sub-tropical broadleaf deciduous forest and cropland during the non-breeding season which may be an important consideration when choosing potential sites to protect.

On a range-wide scale, it appears chats exhibit strong migratory connectivity on a subspecies level; therefore, subspecies-specific conservation planning may be necessary to address population needs. However, within the western subspecies, there appears to be weak migratory connectivity with chats throughout their range overwintering in western Mexico. Therefore, protecting non-breeding habitats in western Mexico would benefit multiple breeding populations for the western subspecies.

Further study of the eastern subspecies is necessary to better understand migratory connectivity patterns within this subspecies. Our research suggests that chats from multiple breeding populations are passing through Chiapas and Veracruz during migration. As others have stated, we recommend continued international partnerships (e.g. this study, Partners in Flight) and the inclusion of diverse stakeholders moving forward to protect and conserve migratory songbirds across their full annual cycle (Nevins et al. 2009; Ortego-Santos et al. 2019).

## Supplementary Information

Below is the link to the electronic supplementary material.

Supplementary file1 (DOCX 113 kb)

Supplementary file2 (DOCX 138 kb)

Supplementary file3 (DOCX 299 kb)

## Data Availability

The datasets supporting the conclusions of this article will be available in the Open Science Framework Repository.

## Code availability

The R code supporting the conclusions of this article will be available in the Open Science Framework Repository.
