# Supplementary material for: Migration and non-breeding ecology of the Yellow-breasted Chat Icteria virens
Source: J Ornithol. 2021 Oct 15;163(1):37–50. doi: 10.1007/s10336-021-01931-8 (PMC8761137; doi:10.1007/s10336-021-01931-8)
Supplement: Supplementary file 1 — Supplementary file1 (DOCX 113 kb) [file 10336_2021_1931_MOESM1_ESM.docx]

**Online Resource 1**

Migration and overwintering ecology of the yellow-breasted chat

Kristen A. Mancuso^*^, Karen E. Hodges, John D. Alexander, Manuel Grosselet, A. Michael Bezener, Luis Morales, Sarahy C. Martinez, Jessica Castellanos-Labarcena, Michael A. Russello, Sarah M. Rockwell, Matthias E. Bieber, Christine A. Bishop

*corresponding author:

[Kmancuso88@gmail.com](mailto:Kmancuso88@gmail.com)

250-864-5788

**Yellow-breasted Chat subspecies genetics determination**

DNA was extracted from the calamus of the outer rectrix feather. Extractions were performed using 100 μL of 5% Chelex®-100 (Bio-Rad laboratories, CA, USA) in ddH_2_0, 5 μL of proteinase K, and 3 μl of DTT and incubated for 4 hours at 56° C. A volume ranging from 80 to 90 μL of DNA extract was removed for further analysis. Initial tests revealed that feather DNA concentrations were too low to genotype the extracts directly. Consequently, we used the polymerase chain reaction to amplify a 1018 nucleotide region of the mtDNA genome spanning the ATPase 8 and ATPase 6 genes along with the complete tRNA-lysine and a short flanking region of the cytochrome oxidase II and III genes using previously published external primers (ATP9947H and ATP8929L; Hunt et al. 2001). Samples were amplified in a total volume of 12.5 µL containing: 6.9 µL of ddH_2_O, 1.25 of dNTPs 2 µM (KAPPA Biosystems), 1.25 µL of forward and reverse primer (10 µM), 1.25 µL of 10X Buffer with MgCl_2_ (Applied Biosystems), 0.1 µL of AmpliTaq Gold DNA polymerase 5U/µL (Applied Biosystems) and 1 µL of DNA. Cycling conditions were as follows: an initial denaturation at 95 °C for 10 minutes followed by 35 cycles of 95 °C for 30 s, 50 °C for 30 s, and 72 °C for 60 s, and a final extension at 72 °C for 7 min.

Previous genetic analyses based on the ATPase gene in the mitochondrial genome identified 18 unique haplotypes within 34 chats sampled from across their North American distribution, including both *Icteria.virens auricollis* *and I.v. virens* (Lovette et al. 2004). Importantly, the authors reconstructed two distinct haplotype groups that were geographically concordant with the breeding distribution of the two subspecies, revealing several single nucleotide polymorphisms (SNPs) that were diagnostic of western and eastern chats. Here, we designed and validated novel TaqMan^®^ genotyping assays targeting diagnostic SNPs in the mitochondrial ATPase gene to rapidly and cost-effectively identify eastern and western chats sampled in their overwintering range. Taqman^®^ genotyping assays detect the two possible variants at a SNP within a target sequence using two fluorescent probes in each reaction. We used previously published mtDNA ATPase sequences from the reference dataset compiled by Lovette et al. (2004) to design two genotyping assays (Iv_ATPase_SNP200; Iv_ATPase_SNP778) using the Custom TaqMan^®^ Assay Design Tool provided by Life Technologies. We initially validated the diagnostic ability of the assays by genotyping 79 reference samples of known locality and subspecies origin across the North American range of *I. virens* as part of the Bird Genoscape Project ([www.birdgenoscape.org](http://www.birdgenoscape.org)): 39 samples of eastern *I.v.* *virens* subspecies (Indiana, Kentucky, Alabama, Missouri) and 40 samples for the western *I.v*. *auricollis* subspecies (Montana, California, Oregon, British Columbia). We then used the Taqman^®^ genotyping assays to identify the subspecies of 211 samples, including 38 from Chiapas, 19 from Nayarit and 154 from Veracruz (Table OR1.1). Note that in Table OR1.1, an additional 10 samples from California and 10 samples from British Columbia were run through the assay but the location was blind to the scientist running the assay. These 20 samples were correctly identified to the western subspecies, further validating the assay.

Genotyping reactions were then performed in a 6 μL final volume, consisting of 2.50 μL of TaqMan^®^ Universal PCR Master Mix, 1.25 μL of ddH2O, 0.25 μL of 20X SNP genotyping assay mix and 2 μL of the PCR product with the amplified ATPase fragment. Non-template controls for each assay were included in each experiment. Reactions were performed on the ViiA™ 7 Real-Time PCR System (Applied Biosystems) with cycling conditions of 60°C for 30 s, 95°C for 10 minutes followed by 40 cycles of 95° C for 15 seconds and 60° C for 1 min, and a final step of 60° C for 30 seconds. Genotype calls were performed with the Applied Biosystems® TaqMan® Genotyper™ Software.

**Figure OR1.1**. Example amplification plot of 48 samples for assay Iv_ATPase_SNP778. Squares represent reference samples and negative control. Dots represent each individual analyzed. Blue symbols represent the eastern *I.v.* *virens* subspecies haplotype, while red symbols represent the western *I.v. auricollis* subspecies haplotype.

**Table OR1.1.** Assay results assigning Yellow-breasted Chat (*Icteria virens*) samples to subspecies. Haplotype at each assay based on nucleotide character state (Iv_ATPase_SNP200 = A or C; Iv_ATPase_SNP778 = G or T), and genetic assignment to subspecies (*auricollis* or *virens*).

| **Sample** | **Type** | **Country** | **State/Province** | **Haplotype** | | **Subspecies** |
| --- | --- | --- | --- | --- | --- | --- |
|  |  |  |  | SNP200 | SNP778 |  |
| 00N5434 | Reference | USA | Oregon | A | C | *auricollis* |
| 00N5435 | Reference | USA | Oregon | A | C | *auricollis* |
| 00N5436 | Reference | USA | Oregon | A | C | *auricollis* |
| 00N5437 | Reference | USA | Oregon | A | C | *auricollis* |
| 00N5438 | Reference | USA | Oregon | A | C | *auricollis* |
| 00N5439 | Reference | USA | Oregon | A | C | *auricollis* |
| 00N5440 | Reference | USA | Oregon | A | C | *auricollis* |
| 01N7492 | Reference | USA | Oregon | A | C | *auricollis* |
| 01N7493 | Reference | USA | Oregon | A | C | *auricollis* |
| 01N7494 | Reference | USA | Oregon | A | C | *auricollis* |
| 03N3464 | Reference | USA | Montana | A | C | *auricollis* |
| 05N5857 | Reference | USA | Montana | A | C | *auricollis* |
| 05N5934 | Reference | USA | Montana | A | C | *auricollis* |
| 06N1697 | Reference | USA | Montana | A | C | *auricollis* |
| 06N1698 | Reference | USA | Montana | A | C | *auricollis* |
| 06N1699 | Reference | USA | Montana | A | C | *auricollis* |
| 06N1700 | Reference | USA | Montana | A | C | *auricollis* |
| 06N24258 | Reference | USA | Montana | A | C | *auricollis* |
| 06N24262 | Reference | USA | Montana | A | C | *auricollis* |
| 06N24272 | Reference | USA | Montana | A | C | *auricollis* |
| 06N25258 | Reference | USA | California | A | C | *auricollis* |
| 06N25259 | Reference | USA | California | A | C | *auricollis* |
| 06N25280 | Reference | USA | California | A | C | *auricollis* |
| 06N25287 | Reference | USA | California | A | C | *auricollis* |
| 06N25288 | Reference | USA | California | A | C | *auricollis* |
| 06N25293 | Reference | USA | California | A | C | *auricollis* |
| 96N4049 | Reference | USA | California | A | C | *auricollis* |
| 96N4051 | Reference | USA | California | A | C | *auricollis* |
| 96N4055 | Reference | USA | California | A | C | *auricollis* |
| 06N25272 | Reference | USA | California | ? | ? | *?* |
| 1321-53909 | Reference | Canada | British Columbia | A | C | *auricollis* |
| 2241-35302 | Reference | Canada | British Columbia | A | C | *auricollis* |
| 2241-3692 | Reference | Canada | British Columbia | A | C | *auricollis* |
| 2241-36924 | Reference | Canada | British Columbia | A | C | *auricollis* |
| 2241-36964 | Reference | Canada | British Columbia | A | C | *auricollis* |
| 2241-36983 | Reference | Canada | British Columbia | A | C | *auricollis* |
| 2241-37000 | Reference | Canada | British Columbia | A | C | *auricollis* |
| 36922 | Reference | Canada | British Columbia | A | C | *auricollis* |
| 36943 | Reference | Canada | British Columbia | A | C | *auricollis* |
| **Sample** | **Type** | **Country** | **State/Province** | **Haplotype** | | **Subspecies** |
|  |  |  |  | SNP200 | SNP778 |  |
| 00N6050 | Reference | USA | Missouri | G | T | *virens* |
| 00N6051 | Reference | USA | Missouri | G | T | *virens* |
| 01N4013 | Reference | USA | Missouri | G | T | *virens* |
| 01N4014 | Reference | USA | Missouri | G | T | *virens* |
| 01N4017 | Reference | USA | Missouri | G | T | *virens* |
| 01N4018 | Reference | USA | Missouri | G | T | *virens* |
| 01N4025 | Reference | USA | Missouri | G | T | *virens* |
| 01N4026 | Reference | USA | Missouri | G | T | *virens* |
| 01N4027 | Reference | USA | Missouri | ? | T | *virens* |
| 00N6049 | Reference | USA | Missouri | G | T | *virens* |
| 04N7437 | Reference | USA | Kentucky | G | T | *virens* |
| 04N7444 | Reference | USA | Kentucky | G | T | *virens* |
| 04N7438 | Reference | USA | Kentucky | G | T | *virens* |
| 04N7439 | Reference | USA | Kentucky | G | T | *virens* |
| 04N7440 | Reference | USA | Kentucky | G | T | *virens* |
| 04N7442 | Reference | USA | Kentucky | G | T | *virens* |
| 05N4365 | Reference | USA | Kentucky | G | T | *virens* |
| 05N4366 | Reference | USA | Kentucky | G | T | *virens* |
| 04N7441 | Reference | USA | Kentucky | G | ? | *virens* |
| 04N7443 | Reference | USA | Kentucky | G | T | *virens* |
| 04N9209 | Reference | USA | Indiana | G | T | *virens* |
| 04N9210 | Reference | USA | Indiana | G | T | *virens* |
| 00N6578 | Reference | USA | Indiana | G | T | *virens* |
| 06N23561 | Reference | USA | Indiana | G | T | *virens* |
| 06N23597 | Reference | USA | Indiana | G | T | *virens* |
| 07N23125 | Reference | USA | Indiana | G | T | *virens* |
| 11N1079 | Reference | USA | Indiana | G | T | *virens* |
| 6N23558 | Reference | USA | Indiana | G | T | *virens* |
| 00N6579 | Reference | USA | Indiana | G | T | *virens* |
| 02N2381 | Reference | USA | Indiana | G | T | *virens* |
| 08N0536 | Reference | USA | Alabama | G | T | *virens* |
| 10N11223 | Reference | USA | Alabama | G | T | *virens* |
| 10N11224 | Reference | USA | Alabama | G | T | *virens* |
| 13N1058 | Reference | USA | Alabama | G | T | *virens* |
| 13N1059 | Reference | USA | Alabama | G | T | *virens* |
| 13N1074 | Reference | USA | Alabama | G | T | *virens* |
| 13N1075 | Reference | USA | Alabama | G | T | *virens* |
| 13N1082 | Reference | USA | Alabama | G | T | *virens* |
| 13N1083 | Reference | USA | Alabama | G | T | *virens* |
| 14N0308 | Reference | USA | Alabama | G | T | *virens* |
| 2241-37972 | Unknown | Canada | British Columbia | A | C | *auricollis* |
| 2421-77218 | Unknown | Canada | British Columbia | A | C | *auricollis* |
| 2541-98099 | Unknown | Canada | British Columbia | A | C | *auricollis* |
| **Sample** | **Type** | **Country** | **State/Province** | **Haplotype** | | **Subspecies** |
|  |  |  |  | SNP200 | SNP778 |  |
| 2561-00119 | Unknown | Canada | British Columbia | A | C | *auricollis* |
| 2561-00213 | Unknown | Canada | British Columbia | A | C | *auricollis* |
| 2561-00215 | Unknown | Canada | British Columbia | A | C | *auricollis* |
| 2561-00216 | Unknown | Canada | British Columbia | A | C | *auricollis* |
| 2561-00230 | Unknown | Canada | British Columbia | A | C | *auricollis* |
| 2561-00260 | Unknown | Canada | British Columbia | A | C | *auricollis* |
| 2561-00262 | Unknown | Canada | British Columbia | A | C | *auricollis* |
| 2711-16058 | Unknown | USA | California | A | C | *auricollis* |
| 2711-16059 | Unknown | USA | California | A | C | *auricollis* |
| 2711-16065 | Unknown | USA | California | A | C | *auricollis* |
| 2711-16066 | Unknown | USA | California | A | C | *auricollis* |
| 2711-16068 | Unknown | USA | California | A | C | *auricollis* |
| 2711-16069 | Unknown | USA | California | A | C | *auricollis* |
| 1951-81015 | Unknown | USA | California | A | C | *auricollis* |
| 1951-81019 | Unknown | USA | California | A | C | *auricollis* |
| 1951-81026 | Unknown | USA | California | A | C | *auricollis* |
| 1951-81023 | Unknown | USA | California | A | C | *auricollis* |
| TDA-D0041563 | Unknown | Mexico | Chiapas | G | T | *virens* |
| TDA-D0041565 | Unknown | Mexico | Chiapas | G | T | *virens* |
| TDA-D0041576 | Unknown | Mexico | Chiapas | G | T | *virens* |
| TDA-D0041565 | Unknown | Mexico | Chiapas | G | T | *virens* |
| TDA-D0041577 | Unknown | Mexico | Chiapas | G | T | *virens* |
| TDA-D0043180 | Unknown | Mexico | Chiapas | G | T | *virens* |
| TDA-D0043330 | Unknown | Mexico | Chiapas | G | T | *virens* |
| TDA-D0043331 | Unknown | Mexico | Chiapas | G | T | *virens* |
| TDA-D0043341 | Unknown | Mexico | Chiapas | G | T | *virens* |
| TDA-D0043345 | Unknown | Mexico | Chiapas | G | T | *virens* |
| TDA-D0043350 | Unknown | Mexico | Chiapas | G | T | *virens* |
| TDA-D0043351 | Unknown | Mexico | Chiapas | G | T | *virens* |
| TDA-D0043352 | Unknown | Mexico | Chiapas | G | T | *virens* |
| TDA-D0043374 | Unknown | Mexico | Chiapas | G | T | *virens* |
| TDA-D0043512 | Unknown | Mexico | Chiapas | G | T | *virens* |
| TDA-D0043514 | Unknown | Mexico | Chiapas | G | T | *virens* |
| TDA-D0043537 | Unknown | Mexico | Chiapas | G | T | *virens* |
| TDA-D0043538 | Unknown | Mexico | Chiapas | G | T | *virens* |
| TDA-D0043541 | Unknown | Mexico | Chiapas | G | T | *virens* |
| TDA-D0043339 | Unknown | Mexico | Chiapas | G | T | *virens* |
| TDA-D0043582 | Unknown | Mexico | Chiapas | G | T | *virens* |
| TDA-D0043568 | Unknown | Mexico | Chiapas | G | T | *virens* |
| TDA-D0043584 | Unknown | Mexico | Chiapas | G | T | *virens* |
| TDA-D0043585 | Unknown | Mexico | Chiapas | G | T | *virens* |
| TDA-D0043587 | Unknown | Mexico | Chiapas | G | T | *virens* |
| TDA-D0043589 | Unknown | Mexico | Chiapas | G | T | *virens* |
| **Sample** | **Type** | **Country** | **State/Province** | **Haplotype** | | **Subspecies** |
|  |  |  |  | SNP200 | SNP778 |  |
| TDA-D0043597 | Unknown | Mexico | Chiapas | G | T | *virens* |
| TDA-D0043598 | Unknown | Mexico | Chiapas | G | T | *virens* |
| TDA-C0035271 | Unknown | Mexico | Chiapas | G | T | *virens* |
| TDA-C0035272 | Unknown | Mexico | Chiapas | G | T | *virens* |
| TDA-C0036008 | Unknown | Mexico | Chiapas | G | T | *virens* |
| TDA-C0036046 | Unknown | Mexico | Chiapas | G | T | *virens* |
| TDA-C0036086 | Unknown | Mexico | Chiapas | G | T | *virens* |
| TDA-C0036099 | Unknown | Mexico | Chiapas | G | T | *virens* |
| TDA-C0036047 | Unknown | Mexico | Chiapas | G | T | *virens* |
| TDA-C0036102 | Unknown | Mexico | Chiapas | G | T | *virens* |
| TDA-D0041559 | Unknown | Mexico | Chiapas | G | T | *virens* |
| No Band | Unknown | Mexico | Chiapas | G | T | *virens* |
| 2241-37935 | Unknown | Mexico | Nayarit | A | C | *auricollis* |
| 2241-37936 | Unknown | Mexico | Nayarit | A | C | *auricollis* |
| 2241-37937 | Unknown | Mexico | Nayarit | A | C | *auricollis* |
| 2241-37938 | Unknown | Mexico | Nayarit | A | C | *auricollis* |
| 2241-37940 | Unknown | Mexico | Nayarit | A | C | *auricollis* |
| 2241-37944 | Unknown | Mexico | Nayarit | A | C | *auricollis* |
| 2241-37948 | Unknown | Mexico | Nayarit | A | C | *auricollis* |
| 2241-37949 | Unknown | Mexico | Nayarit | A | C | *auricollis* |
| 2241-37950 | Unknown | Mexico | Nayarit | A | C | *auricollis* |
| 2241-37952 | Unknown | Mexico | Nayarit | A | C | *auricollis* |
| 2241-37955 | Unknown | Mexico | Nayarit | A | C | *auricollis* |
| 2241-37956 | Unknown | Mexico | Nayarit | A | C | *auricollis* |
| 2241-37958 | Unknown | Mexico | Nayarit | A | C | *auricollis* |
| 2561-00210 | Unknown | Mexico | Nayarit | A | C | *auricollis* |
| 2561-00212 | Unknown | Mexico | Nayarit | A | C | *auricollis* |
| 41105 | Unknown | Mexico | Nayarit | A | C | *auricollis* |
| TDA-C0035412 | Unknown | Mexico | Nayarit | A | C | *auricollis* |
| TDA-C0035413 | Unknown | Mexico | Nayarit | A | C | *auricollis* |
| TDA-C0035414 | Unknown | Mexico | Nayarit | A | C | *auricollis* |
| TDA-D0021882 | Unknown | Mexico | Veracruz | G | T | *virens* |
| TDA-D0021887 | Unknown | Mexico | Veracruz | G | T | *virens* |
| TDA-D0021889 | Unknown | Mexico | Veracruz | G | T | *virens* |
| TDA-D0021894 | Unknown | Mexico | Veracruz | G | T | *virens* |
| TDA-D0021910 | Unknown | Mexico | Veracruz | G | T | *virens* |
| TDA-D0021976 | Unknown | Mexico | Veracruz | G | T | *virens* |
| TDA-D0021979 | Unknown | Mexico | Veracruz | G | T | *virens* |
| TDA-D0021984 | Unknown | Mexico | Veracruz | G | T | *virens* |
| TDA-D0021987 | Unknown | Mexico | Veracruz | G | T | *virens* |
| TDA-D0021992 | Unknown | Mexico | Veracruz | G | T | *virens* |
| TDA-D0022004 | Unknown | Mexico | Veracruz | ? | ? | *?* |
| TDA-D0022030 | Unknown | Mexico | Veracruz | G | T | *virens* |
| **Sample** | **Type** | **Country** | **State/Province** | **Haplotype** | | **Subspecies** |
|  |  |  |  | SNP200 | SNP778 |  |
| TDA-D0022043 | Unknown | Mexico | Veracruz | G | T | *virens* |
| TDA-D0022047 | Unknown | Mexico | Veracruz | G | T | *virens* |
| TDA-D0022061 | Unknown | Mexico | Veracruz | G | T | *virens* |
| TDA-D0022074 | Unknown | Mexico | Veracruz | G | T | *virens* |
| TDA-D0022076 | Unknown | Mexico | Veracruz | G | T | *virens* |
| TDA-D0022160 | Unknown | Mexico | Veracruz | G | T | *virens* |
| TDA-D0022233 | Unknown | Mexico | Veracruz | G | T | *virens* |
| TDA-D0022239 | Unknown | Mexico | Veracruz | G | T | *virens* |
| TDA-D0022241 | Unknown | Mexico | Veracruz | ? | ? | *?* |
| TDA-D0022294 | Unknown | Mexico | Veracruz | G | T | *virens* |
| TDA-D0022300 | Unknown | Mexico | Veracruz | G | T | *virens* |
| TDA-D0022323 | Unknown | Mexico | Veracruz | G | T | *virens* |
| TDA-D0022422 | Unknown | Mexico | Veracruz | G | T | *virens* |
| TDA-D0022600 | Unknown | Mexico | Veracruz | G | T | *virens* |
| TDA-D0022860 | Unknown | Mexico | Veracruz | G | T | *virens* |
| TDA-D0022987 | Unknown | Mexico | Veracruz | G | T | *virens* |
| TDA-D0023061 | Unknown | Mexico | Veracruz | G | T | *virens* |
| TDA-D0023065 | Unknown | Mexico | Veracruz | G | T | *virens* |
| TDA-D0023336 | Unknown | Mexico | Veracruz | G | T | *virens* |
| TDA-D0023474 | Unknown | Mexico | Veracruz | G | T | *virens* |
| TDA-D0023475 | Unknown | Mexico | Veracruz | G | T | *virens* |
| TDA-D0023476 | Unknown | Mexico | Veracruz | G | T | *virens* |
| TDA-D0023479 | Unknown | Mexico | Veracruz | G | T | *virens* |
| TDA-D0023537 | Unknown | Mexico | Veracruz | G | T | *virens* |
| TDA-D0023539 | Unknown | Mexico | Veracruz | G | T | *virens* |
| TDA-D0023550 | Unknown | Mexico | Veracruz | G | ? | *virens* |
| TDA-D0023567 | Unknown | Mexico | Veracruz | G | T | *virens* |
| TDA-D0023571 | Unknown | Mexico | Veracruz | G | T | *virens* |
| TDA-D0023598 | Unknown | Mexico | Veracruz | G | T | *virens* |
| TDA-D0024216 | Unknown | Mexico | Veracruz | G | T | *virens* |
| TDA-D0024223 | Unknown | Mexico | Veracruz | G | T | *virens* |
| TDA-D0024268 | Unknown | Mexico | Veracruz | G | T | *virens* |
| TDA-D0024271 | Unknown | Mexico | Veracruz | G | ? | *virens* |
| TDA-D0024281 | Unknown | Mexico | Veracruz | G | T | *virens* |
| TDA-D0024285 | Unknown | Mexico | Veracruz | G | T | *virens* |
| TDA-D0024291 | Unknown | Mexico | Veracruz | G | T | *virens* |
| TDA-D0024700 | Unknown | Mexico | Veracruz | G | T | *virens* |
| TDA-D0024701 | Unknown | Mexico | Veracruz | G | T | *virens* |
| TDA-D0024703 | Unknown | Mexico | Veracruz | G | T | *virens* |
| TDA-D0024710 | Unknown | Mexico | Veracruz | G | T | *virens* |
| TDA-D0024983 | Unknown | Mexico | Veracruz | G | T | *virens* |
| TDA-D0024991 | Unknown | Mexico | Veracruz | G | T | *virens* |
| TDA-D0024992 | Unknown | Mexico | Veracruz | G | T | *virens* |
| **Sample** | **Type** | **Country** | **State/Province** | **Haplotype** | | **Subspecies** |
|  |  |  |  | SNP200 | SNP778 |  |
| TDA-D0024993 | Unknown | Mexico | Veracruz | G | T | *virens* |
| TDA-D0024997 | Unknown | Mexico | Veracruz | G | T | *virens* |
| TDA-D0024998 | Unknown | Mexico | Veracruz | G | T | *virens* |
| TDA-D0024999 | Unknown | Mexico | Veracruz | G | T | *virens* |
| TDA-D0025028 | Unknown | Mexico | Veracruz | G | T | *virens* |
| TDA-D0025029 | Unknown | Mexico | Veracruz | G | T | *virens* |
| TDA-D0025036 | Unknown | Mexico | Veracruz | G | T | *virens* |
| TDA-D0025048 | Unknown | Mexico | Veracruz | G | T | *virens* |
| TDA-D0025056 | Unknown | Mexico | Veracruz | ? | ? | *?* |
| TDA-D0025200 | Unknown | Mexico | Veracruz | G | T | *virens* |
| TDA-D0025256 | Unknown | Mexico | Veracruz | G | T | *virens* |
| TDA-D0025258 | Unknown | Mexico | Veracruz | G | T | *virens* |
| TDA-D0025260 | Unknown | Mexico | Veracruz | G | T | *virens* |
| TDA-D0025264 | Unknown | Mexico | Veracruz | G | T | *virens* |
| TDA-D0025268 | Unknown | Mexico | Veracruz | G | T | *virens* |
| TDA-D0025271 | Unknown | Mexico | Veracruz | G | T | *virens* |
| TDA-D0025335 | Unknown | Mexico | Veracruz | ? | ? | *?* |
| TDA-D0025339 | Unknown | Mexico | Veracruz | G | T | *virens* |
| TDA-D0025399 | Unknown | Mexico | Veracruz | G | T | *virens* |
| TDA-D0025411 | Unknown | Mexico | Veracruz | G | T | *virens* |
| TDA-D0025412 | Unknown | Mexico | Veracruz | ? | ? | *?* |
| TDA-D0025420 | Unknown | Mexico | Veracruz | G | T | *virens* |
| TDA-D0025423 | Unknown | Mexico | Veracruz | G | T | *virens* |
| TDA-D0025425 | Unknown | Mexico | Veracruz | G | T | *virens* |
| TDA-D0025428 | Unknown | Mexico | Veracruz | G | T | *virens* |
| TDA-D0025497 | Unknown | Mexico | Veracruz | G | T | *virens* |
| TDA-D0025509 | Unknown | Mexico | Veracruz | ? | T | *virens* |
| TDA-D0025528 | Unknown | Mexico | Veracruz | G | T | *virens* |
| TDA-D0025539 | Unknown | Mexico | Veracruz | G | T | *virens* |
| TDA-D0025550 | Unknown | Mexico | Veracruz | G | T | *virens* |
| TDA-D0025551 | Unknown | Mexico | Veracruz | G | T | *virens* |
| TDA-D0025561 | Unknown | Mexico | Veracruz | G | T | *virens* |
| TDA-D0025577 | Unknown | Mexico | Veracruz | G | T | *virens* |
| TDA-D0025579 | Unknown | Mexico | Veracruz | G | T | *virens* |
| TDA-D0025585 | Unknown | Mexico | Veracruz | G | T | *virens* |
| TDA-D0025588 | Unknown | Mexico | Veracruz | G | T | *virens* |
| TDA-D0025601 | Unknown | Mexico | Veracruz | ? | ? | *?* |
| TDA-D0025605 | Unknown | Mexico | Veracruz | G | T | *virens* |
| TDA-D0025626 | Unknown | Mexico | Veracruz | G | T | *virens* |
| TDA-D0025634 | Unknown | Mexico | Veracruz | G | T | *virens* |
| TDA-D0025641 | Unknown | Mexico | Veracruz | G | T | *virens* |
| TDA-D0025651 | Unknown | Mexico | Veracruz | ? | ? | *?* |
| TDA-D0025652 | Unknown | Mexico | Veracruz | G | T | *virens* |
| **Sample** | **Type** | **Country** | **State/Province** | **Haplotype** | | **Subspecies** |
|  |  |  |  | SNP200 | SNP778 |  |
| TDA-D0025654 | Unknown | Mexico | Veracruz | G | T | *virens* |
| TDA-D0025681 | Unknown | Mexico | Veracruz | G | T | *virens* |
| TDA-D0025682 | Unknown | Mexico | Veracruz | G | T | *virens* |
| TDA-D0025684 | Unknown | Mexico | Veracruz | G | T | *virens* |
| TDA-D0025688 | Unknown | Mexico | Veracruz | G | T | *virens* |
| TDA-D0025691 | Unknown | Mexico | Veracruz | ? | ? | *?* |
| TDA-D0025697 | Unknown | Mexico | Veracruz | G | T | *virens* |
| TDA-D0025702 | Unknown | Mexico | Veracruz | G | T | *virens* |
| TDA-D0025849 | Unknown | Mexico | Veracruz | G | T | *virens* |
| TDA-D0025851 | Unknown | Mexico | Veracruz | ? | ? | *?* |
| TDA-D002589 | Unknown | Mexico | Veracruz | G | T | *virens* |
| TDA-D0025919 | Unknown | Mexico | Veracruz | G | T | *virens* |
| TDA-D0025937 | Unknown | Mexico | Veracruz | G | T | *virens* |
| TDA-D0026008 | Unknown | Mexico | Veracruz | G | T | *virens* |
| TDA-D0026010 | Unknown | Mexico | Veracruz | G | T | *virens* |
| TDA-D0026087 | Unknown | Mexico | Veracruz | G | T | *virens* |
| TDA-D0026088 | Unknown | Mexico | Veracruz | G | T | *virens* |
| TDA-D0026152 | Unknown | Mexico | Veracruz | G | T | *virens* |
| TDA-D0026155 | Unknown | Mexico | Veracruz | G | T | *virens* |
| TDA-D0026156 | Unknown | Mexico | Veracruz | G | T | *virens* |
| TDA-D0026183 | Unknown | Mexico | Veracruz | G | T | *virens* |
| TDA-D0026194 | Unknown | Mexico | Veracruz | G | T | *virens* |
| TDA-D0026199 | Unknown | Mexico | Veracruz | G | T | *virens* |
| TDA-D0026201 | Unknown | Mexico | Veracruz | G | T | *virens* |
| TDA-D0026210 | Unknown | Mexico | Veracruz | G | T | *virens* |
| TDA-D0026219 | Unknown | Mexico | Veracruz | G | T | *virens* |
| TDA-D0026237 | Unknown | Mexico | Veracruz | G | T | *virens* |
| TDA-D0026241 | Unknown | Mexico | Veracruz | G | T | *virens* |
| TDA-D0026243 | Unknown | Mexico | Veracruz | G | T | *virens* |
| TDA-D0026253 | Unknown | Mexico | Veracruz | G | T | *virens* |
| TDA-D0026260 | Unknown | Mexico | Veracruz | G | T | *virens* |
| TDA-D0026261 | Unknown | Mexico | Veracruz | G | T | *virens* |
| TDA-D0026306 | Unknown | Mexico | Veracruz | G | T | *virens* |
| TDA-D0026335 | Unknown | Mexico | Veracruz | G | T | *virens* |
| TDA-D0026589 | Unknown | Mexico | Veracruz | ? | ? | *?* |
| TDA-D0026649 | Unknown | Mexico | Veracruz | G | T | *virens* |
| TDA-D0026802 | Unknown | Mexico | Veracruz | G | T | *virens* |
| TDA-D0026901 | Unknown | Mexico | Veracruz | G | T | *virens* |
| TDA-D0027279 | Unknown | Mexico | Veracruz | G | T | *virens* |
| TDA-D0027399 | Unknown | Mexico | Veracruz | G | T | *virens* |
| TDA-D0027470 | Unknown | Mexico | Veracruz | G | T | *virens* |
| TDA-D0027476 | Unknown | Mexico | Veracruz | G | T | *virens* |
| TDA-D0027477 | Unknown | Mexico | Veracruz | G | T | *virens* |
| **Sample** | **Type** | **Country** | **State/Province** | **Haplotype** | | **Subspecies** |
|  |  |  |  | SNP200 | SNP778 |  |
| TDA-D0027797 | Unknown | Mexico | Veracruz | G | T | *virens* |
| TDA-D0028580 | Unknown | Mexico | Veracruz | G | T | *virens* |
| TDA-D0028585 | Unknown | Mexico | Veracruz | G | T | *virens* |
| TDA-D0028606 | Unknown | Mexico | Veracruz | G | T | *virens* |
| TDA-D0028609 | Unknown | Mexico | Veracruz | ? | ? | *?* |
| TDA-D0029553 | Unknown | Mexico | Veracruz | G | T | *virens* |
| TDA-D0030303 | Unknown | Mexico | Veracruz | G | T | *virens* |
| TDA-D0030329 | Unknown | Mexico | Veracruz | G | T | *virens* |
| TDA-D0030360 | Unknown | Mexico | Veracruz | G | T | *virens* |
| TDA-D0030358 | Unknown | Mexico | Veracruz | G | T | *virens* |
| TDA-D0025500 | Unknown | Mexico | Veracruz | G | T | *virens* |
| TDA-D0022847 | Unknown | Mexico | Veracruz | G | T | *virens* |

**References:**

Hunt JS, Bermingham E, Ricklefs RE (2001) Molecular systematics and biogeography of antillean thrashers, tremblers, and mockingbirds (aves: Mimidae). Auk 118:35–55. https://doi.org/10.2307/4089757

Lovette IJ, Clegg SM, Smith TB (2004) Limited utility of mtDNA markers for determining connectivity among breeding and overwintering locations in three neotropical migrant birds. Conserv Biol 18:156–166. https://doi.org/10.1111/j.1523-1739.2004.00239.x
