# Supplementary material for: Migration and non-breeding ecology of the Yellow-breasted Chat Icteria virens
Source: J Ornithol. 2021 Oct 15;163(1):37–50. doi: 10.1007/s10336-021-01931-8 (PMC8761137; doi:10.1007/s10336-021-01931-8)
Supplement: Supplementary file 2 — Supplementary file2 (DOCX 138 kb) [file 10336_2021_1931_MOESM2_ESM.docx]

**Online Resource 2**

Migration and overwintering ecology of the yellow-breasted chat

Kristen A. Mancuso^*^, Karen E. Hodges, John D. Alexander, Manuel Grosselet, A. Michael Bezener, Luis Morales, Sarahy C. Martinez, Jessica Castellanos-Labarcena, Michael A. Russello, Sarah M. Rockwell, Matthias E. Bieber, Christine A. Bishop

*corresponding author:

[Kmancuso88@gmail.com](mailto:Kmancuso88@gmail.com)

250-864-5788

**Yellow-breasted Chat hydrogen isotope analyses**

Calibration of the North American precipitation isoscape to reflect stable hydrogen isotope values in Yellow-breasted Chat feathers (*δ*^2^H_f)_. Here we report the samples of known origin to calibrate the isoscape, the plot of the calibration model, the model output, and a visualization of the calibrated isoscape in North America. Note that the 9 values from Hobson et al. (2012) were measured using old calibration values of standard reference materials, and we updated the values using the equation provided in Soto et al. (2017): *δ*^2^H_f_ = 10.774 + 0.852 * old data.

**Table OR2.1**. Known origin Yellow-breasted Chat (*Icteria virens*) *δ*^2^H_f_ values. These values were used to calibrate a precipitation isoscape into a Yellow-breasted Chat feather isoscape. Age codes are after second year (ASY), second year (SY), hatch year (HY), or after hatch year (AHY). Dashes indicate no data. Data shown from Hobson et al. (2012) are transformed values based on the latest calibration methods from Soto et al. (2017).

| **Province or State** | **Longitude** | **Latitude** | **Elevation**  **(m ASL)** | **Sample ID** | **Age** | **Sex** | ***δ*^2^H_f_ value (‰)** | **Source** |
| --- | --- | --- | --- | --- | --- | --- | --- | --- |
| BC | -119.613 | 49.485 | 342 | 2561-00163 | ASY | M | -93.5 | This study |
| BC | -119.552 | 49.200 | 300 | 2561-00262 | SY | M | -91.9 | This study |
| BC | -119.541 | 49.306 | 329 | 2561-00230 | SY | M | -92.3 | This study |
| BC | -119.546 | 49.221 | 303 | 2561-00221 | ASY | M | -80.4 | This study |
| BC | -119.546 | 49.221 | 303 | 2561-00223 | SY | M | -87.5 | This study |
| BC | -119.544 | 49.096 | 286 | 2561-00217 | ASY | M | -83.1 | This study |
| BC | -119.551 | 49.221 | 303 | 2561-00119 | ASY | M | -80.9 | This study |
| BC | -119.544 | 49.096 | 286 | 2561-00219 | ASY | M | -93.7 | This study |
| BC | -119.544 | 49.096 | 286 | 2561-00215 | ASY | M | -81.0 | This study |
| BC | -119.544 | 49.096 | 286 | 2561-00220 | ASY | M | -56.7 | This study |
| BC | -119.551 | 49.221 | 303 | 2241-35320 | SY | F | -88.1 | This study |
| BC | -119.541 | 49.306 | 329 | 2241-35328 | ASY | M | -88.4 | This study |
| BC | -119.552 | 49.200 | 300 | 2691-79842 | SY | F | -87.2 | This study |
| BC | -119.613 | 49.485 | 342 | 2241-37991 | ASY | M | -63.4 | This study |
| BC | -119.551 | 49.221 | 303 | 2421-77218 | ASY | M | -63.3 | This study |
| CA | -122.854 | 40.695 | 534 | 1951-81015 | SY | M | -61.6 | This study |
| CA | -122.854 | 40.695 | 534 | 1951-81017 | SY | M | -60.0 | This study |
| CA | -122.854 | 40.695 | 534 | 2711-16061 | SY | M | -59.1 | This study |
| CA | -122.854 | 40.695 | 534 | 2711-16062 | ASY | M | -63.5 | This study |
| CA | -123.047 | 40.707 | 455 | 1951-81019 | ASY | M | -80.0 | This study |
| CA | -123.047 | 40.707 | 455 | 2711-16068 | SY | M | -65.3 | This study |
| CA | -123.028 | 40.683 | 462 | 2711-16058 | SY | M | -67.3 | This study |
| CA | -122.817 | 40.709 | 552 | 2711-16064 | ASY | M | -65.9 | This study |
| CA | -122.817 | 40.709 | 552 | 1951-81018 | SY | M | -56.7 | This study |
| CA | -122.854 | 40.695 | 534 | 2711-16060 | SY | F | -87.5 | This study |
| CA | -123.028 | 40.683 | 462 | 2711-16070 | SY | F | -58.7 | This study |
| CA | -122.854 | 40.695 | 534 | 2711-16063 | SY | F | -64.5 | This study |
| CA | -123.047 | 40.707 | 455 | 2711-16067 | ASY | F | -57.9 | This study |
| CA | -122.836 | 40.717 | 548 | 2711-16072 | ASY | M | -68.6 | This study |
| CA | -123.028 | 40.683 | 462 | 1951-81025 | ASY | F | -63.6 | This study |
| OR | -122.140 | 42.019 | 905 | 1801-51176 | HY | U | -70.4 | This study |
| OR | -122.140 | 42.019 | 905 | 2741-57607 | HY | U | -76.7 | This study |
| OR | -123.480 | 42.490 | 251 | 2471-12125 | SY | M | -35.1 | This study |
| OR | -123.480 | 42.490 | 251 | 2741-57719 | HY | U | -65.0 | This study |
| OR | -123.480 | 42.490 | 251 | 2741-57997 | AHY | F | -63.4 | This study |
| OR | -123.480 | 42.490 | 251 | 2741-57720 | SY | F | -45.7 | This study |
| OR | -123.480 | 42.490 | 251 | 2741-57993 | SY | F | -53.6 | This study |
| **Province or State** | **Longitude** | **Latitude** | **Elevation**  **(m ASL)** | **Sample ID** | **Age** | **Sex** | **δ^2^H_f_ value (‰)** | **Source** |
| OR | -123.480 | 42.490 | 251 | 2741-57886 | HY | U | -58.4 | This study |
| OR | -123.480 | 42.490 | 251 | 2741-57994 | SY | M | -65.1 | This study |
| OR | -123.480 | 42.490 | 251 | 2741-57995 | SY | M | -36.9 | This study |
| IN | -85.458 | 38.966 | 259 | 454 | - | - | -38.3 | Hobson et al. 2012 |
| IN | -85.380 | 38.860 | 267 | 457 | - | - | -44.1 | Hobson et al. 2012 |
| MI | -86.519 | 33.762 | 80 | 455 | - | - | -25.7 | Hobson et al. 2012 |
| MI | -92.111 | 37.694 | 330 | 458 | - | - | -38.0 | Hobson et al. 2012 |
| MO | -92.180 | 37.779 | 255 | 456 | - | - | -34.6 | Hobson et al. 2012 |
| MO | -92.180 | 37.779 | 255 | 459 | - | - | -38.6 | Hobson et al. 2012 |
| MO | -92.180 | 37.779 | 255 | 460 | - | - | -34.6 | Hobson et al. 2012 |
| MT | -111.915 | 46.934 | 1155 | 461 | - | - | -92.1 | Hobson et al. 2012 |
| OR | -123.480 | 42.490 | 251 | 462 | - | - | -62.7 | Hobson et al. 2012 |


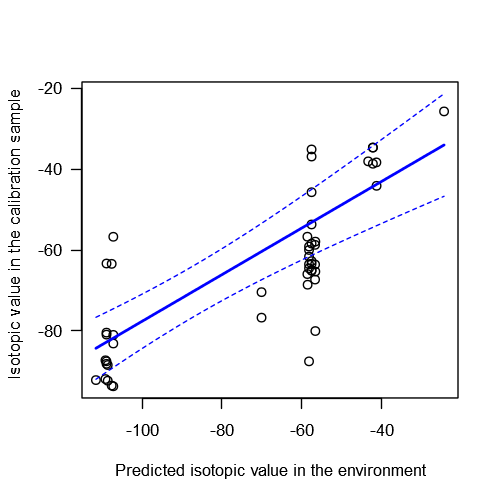


**Figure OR2.1 -** Calibration linear mixed-effects model of *δ*^2^H_f_ observed vs. *δ*^2^H_p_ predicted. The regression line and 95% confidence interval are displayed. This calibration model relates the hydrogen isotopic value in Yellow-breasted Chat (*Icteria virens*) feathers (*δ*^2^H_f_) to the hydrogen isotopic value from precipitation (*δ*^2^H_p_) in the environment. Isotope values are presented per thousand (‰) standardized to Vienna Standard Mean Ocean Water. The calibration samples used to fit the equation are from Table OR2.1. The fit of this model was used to create the Yellow-breasted Chat feather isoscape, which was used to predict the origin of unknown samples.

The linear mixed-effects model calibrating the feather isoscape from the precipitation isoscape included a slope (± SE) of 0.58 ± 0.09 ‰*δ*^2^H_f_/‰*δ*^2^H_p_ and an intercept (± SE) of -19.97 ± 8.43 ‰^2^H_f_.

Model Output:


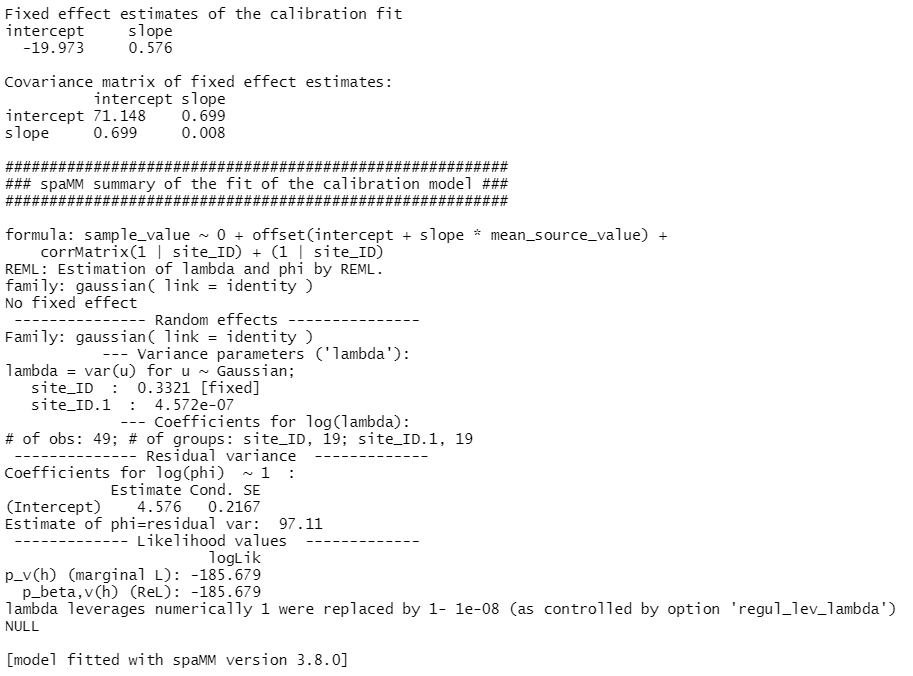


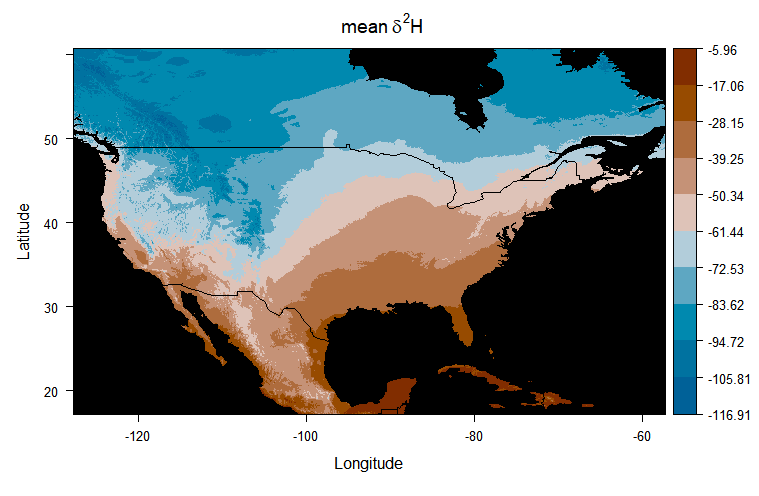


**Figure OR2.2** - Calibrated Yellow-breasted Chat (*Icteria virens*) feather isoscape. Visualization of a precipitation stable hydrogen isoscape that has been calibrated to fit stable hydrogen isotope ratios from Yellow-breasted Chat feathers of known origin. This chat feather isoscape shows the mean stable hydrogen isotope values in feathers across North America and was used during the process of inferring geographic assignment probabilities of chat feathers of unknown origin.


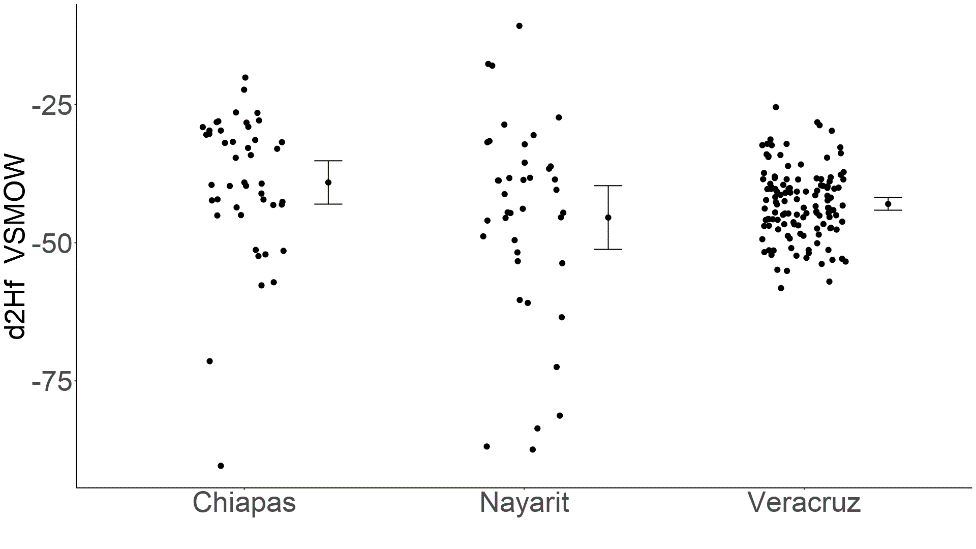
**Figure OR2.3** - Stable hydrogen isotope ratios in Yellow-breasted Chat (*Icteria virens)* feathers (δ^2^H_f_). Stable hydrogen isotope values are reported per thousand (‰) standardized to Vienna Standard Mean Ocean Water (VSMOW). Chats were sampled in Chiapas, Mexico during fall migration from September to October in 2018 (n=45). Chats were sampled in Nayarit, Mexico during the overwintering period in 2018 (n=40). Chats were sampled in Veracruz, Mexico during migration in 2014 (n=125). Stable hydrogen isotope ratios represent that of the breeding ground locations where the feather was grown the previous season. Errors bars represent mean and 95% confidence intervals.

**References:**

Hobson KA, Van Wilgenburg SL, Wassenaar LI, Larson K (2012) Linking hydrogen (δ2H) isotopes in feathers and precipitation: sources of variance and consequences for assignment to isoscapes. PLoS One 7:e35137. https://doi.org/10.1371/journal.pone.0035137

Soto DX, Koehler G, Wassenaar LI, Hobson KA (2017) Re-evaluation of the hydrogen stable isotopic composition of keratin calibration standards for wildlife and forensic science applications. Rapid Commun Mass Spectrom 31:1193–1203. https://doi.org/10.1002/rcm.7893
