# Supplementary material for: Migration and non-breeding ecology of the Yellow-breasted Chat Icteria virens
Source: J Ornithol. 2021 Oct 15;163(1):37–50. doi: 10.1007/s10336-021-01931-8 (PMC8761137; doi:10.1007/s10336-021-01931-8)
Supplement: Supplementary file 3 — Supplementary file3 (DOCX 299 kb) [file 10336_2021_1931_MOESM3_ESM.docx]

**Online Resource 3**

Migration and overwintering ecology of the yellow-breasted chat

Kristen A. Mancuso^*^, Karen E. Hodges, John D. Alexander, Manuel Grosselet, A. Michael Bezener, Luis Morales, Sarahy C. Martinez, Jessica Castellanos-Labarcena, Michael A. Russello, Sarah M. Rockwell, Matthias E. Bieber, Christine A. Bishop

*corresponding author:

[Kmancuso88@gmail.com](mailto:Kmancuso88@gmail.com)

250-864-5788

Yellow-breasted chat stopover site


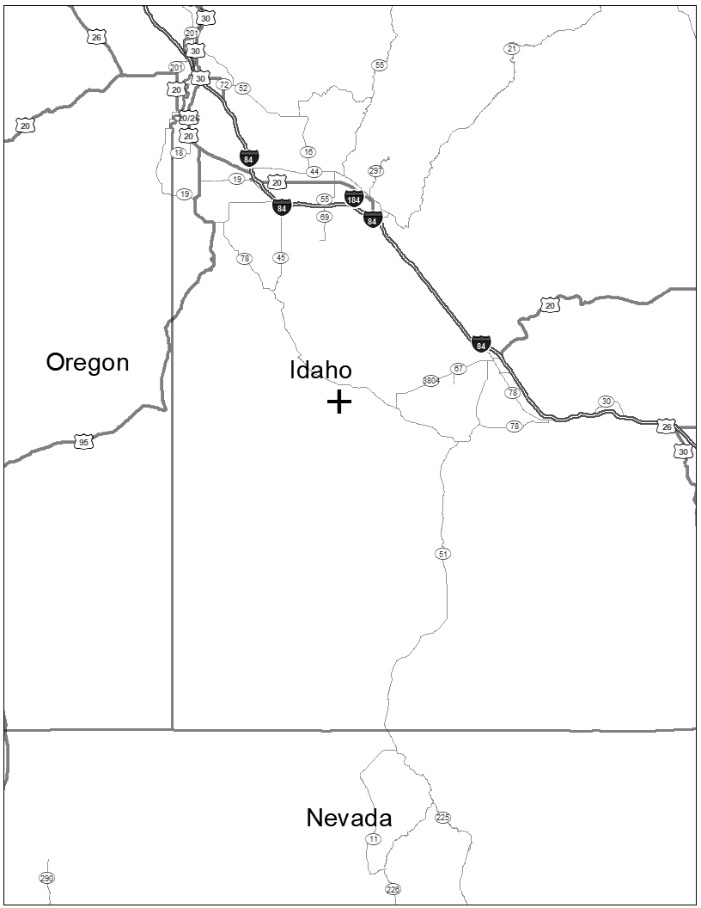

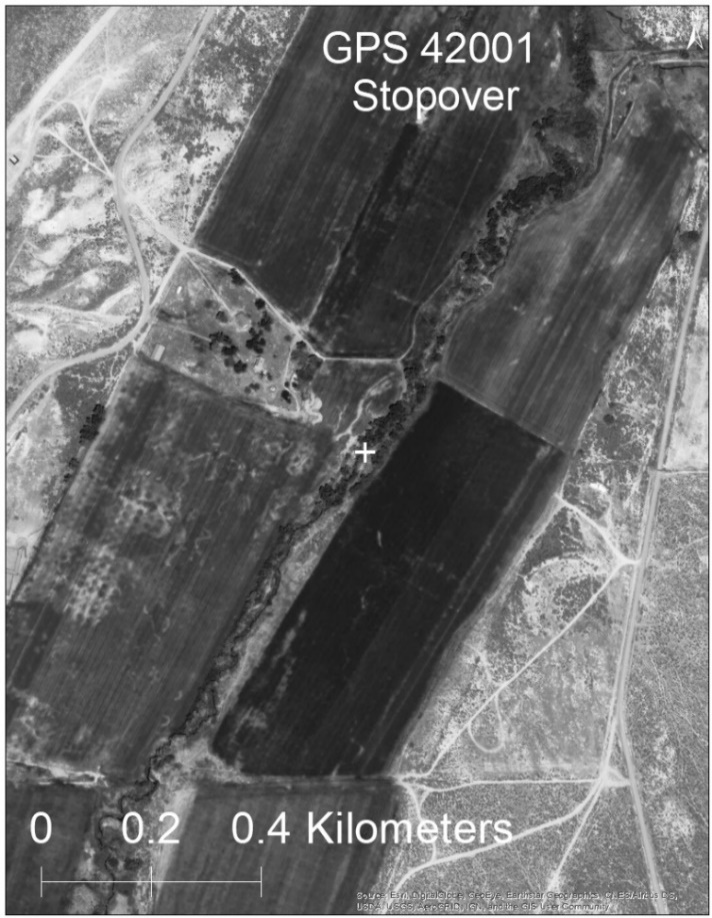


Western yellow-breasted chat (*Icteria virens auricollis*) fall migration stopover site. One Yellow-breasted Chat tracked with a Lotek GPS PinPoint from its breeding site in British Columbia, Canada, stopped during fall migration in Idaho, USA for at least 8 days between September 14 - 22, 2017. The cross marks the stopover site.
